# Digestate biochar effects on soil Pb bioaccessibility, crop Pb concentrations, and human health risk in urban vegetable agroecosystems

**DOI:** 10.1007/s10653-026-03269-7

**Published:** 2026-05-28

**Authors:** Jennifer Newell, Rory Doherty, Gary Lyons, Siobhan F. Cox

**Affiliations:** 1 School of Natural and Built Environment, Queen’s University Belfast, Belfast, BT9 5AG UK; 2 Agri-Environment Branch, Agri-Food and Biosciences Institute, Large Park, Hillsborough, BT26 6DR UK

**Keywords:** Biochar, Urban, Soil, Vegetable, Risk

## Abstract

Biochar is an effective method of Potentially Toxic Element (PTE) remediation in urban agricultural systems; however, its remediation potential may be determined by the extent to which it alters soil properties. Where soil properties are minimally changed with biochar application, remediation potential may be minimally effective. A controlled experiment was used to compare the effects of 5%w/w digestate biochar in no-crop, lettuce, carrot, and garlic agrisystems with differing soil Pb concentrations, high soil pH and organic matter. Total soil and vegetable Pb and bioaccessible soil Pb were quantified using acid digestions and Unified BARGE Bioaccessibility Methodology (UBM). Risk indices (ADI, THQ, CR), were used to assess grower (soil), consumer (vegetable) and combined (soil and vegetable) Pb ingestion risks for each vegetable agrisystem, and were supported by Monte Carlo and sensitivity analyses. Digestate biochar significantly reduced soil-derived Pb ingestion risks (ADI/THQ/CR) in high Pb no-crop systems, high Pb and low Pb carrot systems, and low-Pb garlic systems, though had no effect on ingestion risk in low Pb no-crop and high Pb garlic agrisystems. Notably, biochar did not affect vegetable-derived Pb ingestion risks for any agrisystem. When soil and vegetable risk indices were combined, biochar only lowered overall Pb ingestion risks in high-Pb no-crop and low-Pb lettuce systems, with minimal or inconsistent effects elsewhere. Ultimately, biochar had limited and variable effects on reducing Pb ingestion risks in the studied vegetable agrisystems, which suggests that its remediation potential in urban agrisystems is context-dependent and may offer limited protection for growers and consumers in soils with elevated pH and organic matter.

**Graphical abstract:**

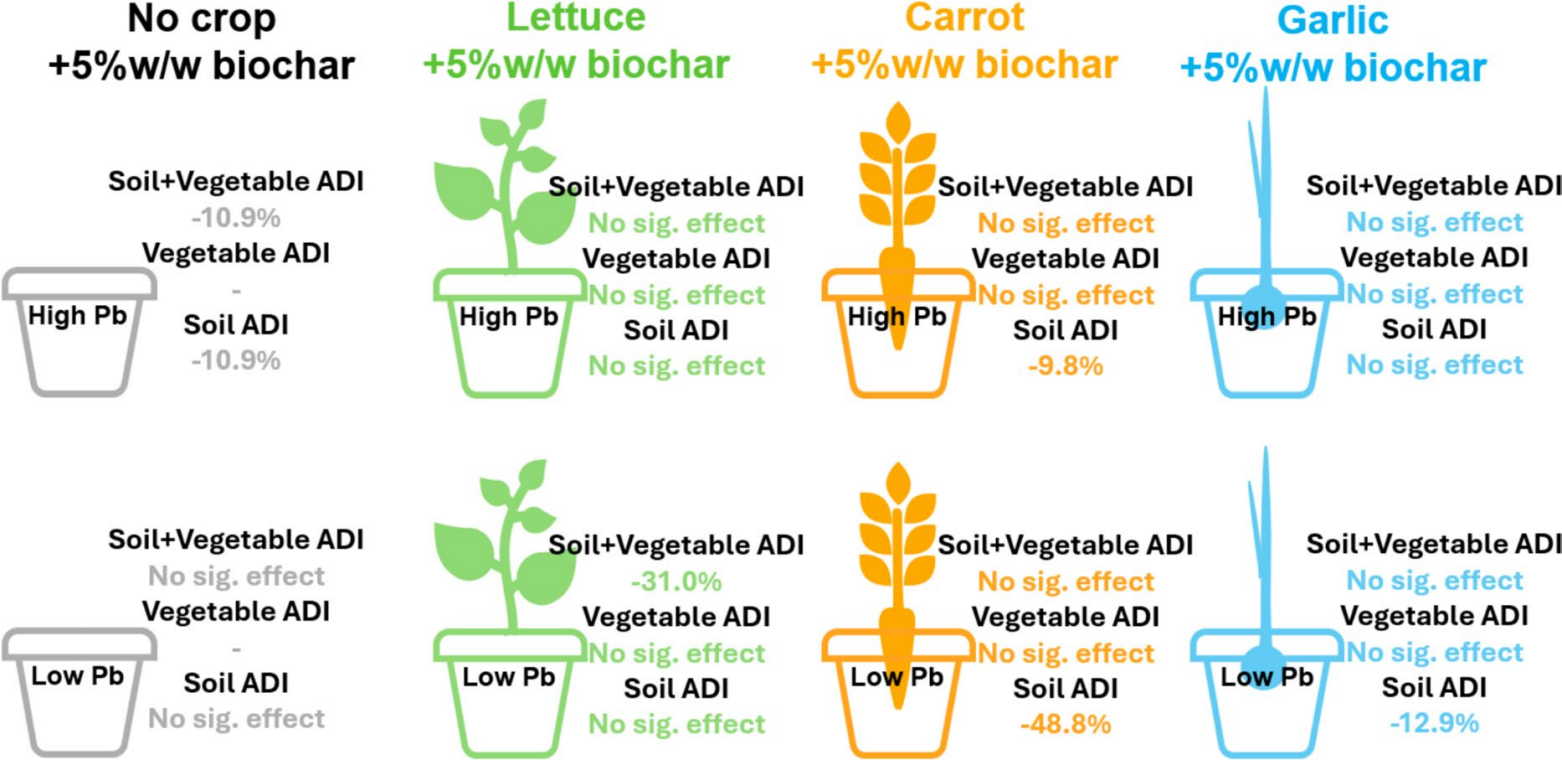

**Supplementary Information:**

The online version contains supplementary material available at 10.1007/s10653-026-03269-7.

## Introduction

The United Nations anticipates a significant increase in the urban population, from 25% of the global population residing in urban areas in 1950, to ~ 68% in 2050 (United Nations, 2019). With this localised increase in population, comes a corresponding expansion of urban infrastructure, increased demand for food and resources, increased waste production, and consequent degradation of environmental, social, and economic welfare (United Nations, 2019).

There has been increasing interest and investment in urban agriculture to meet nutritional demands, with an estimated < 20% of global produce sourced from urban spaces as of 2024 (Payen et al., 2022). Urban crop quality and yield are typically a product of local soil quality however, and urban soils are often poor quality, with high levels of anthropogenic contaminants such as lead, zinc, copper, and other trace metals (Müller et al., 2020). Of these contaminants, lead (Pb) is frequently observed in global soil studies, with a range of reported concentrations including low 0.8 mg/kg in Poland (Mousavi et al., 2022) and high 190,980 mg/kg in North America (Mielke et al., 2000).

From these high soil concentrations, bioavailable soil Pb may enter crop tissue through leaves after atmospheric fallout, or, more commonly, through root uptake from the soil (Fahr et al., 2013). Plant root uptake and Pb phytoaccumulation is dependent on soil Pb mobility and on plant characteristics. *Brassicas* are known hyperaccumulators and can tolerate high tissue Pb concentrations, other plants complex Pb in root structures thus may pose greater ingestion risks in subterranean crops (Pourrut et al., 2011).

After soil ingestion by adult growers and crop ingestion by consumers, bioaccessible Pb (the proportion of Pb that is soluble under gastrointestinal conditions and available for absorption into the circulatory system; Paustenbach (2000)), is absorbed in the intestines and accumulates in the kidneys, liver, and bone, with a residence time from 35 days to up to 30 years (Kumar et al., 2020). Pb toxicity in children manifests as impaired mental development and hyperactivity, and in adults as neuropsychiatric effects under acute exposure, and gastrointestinal issues under chronic exposure (Wani et al., 2015).

Ultimately Pb exposure accounts for ~ 1% of the global disease burden (Ritchie & Roser, 2022). Thus, high soil Pb contamination can be viewed as a major constraint on the development of urban agriculture and Nature-based Solutions (NBS) as it may pose considerable health risks to community growers (Biasioli et al., 2007).

As Pb mobility is known to be affected by soil conditions, soil amendments may be used to reduce potentially high bioaccessibility onsite. In agroecological practices often utilised in urban allotments and community gardens, organic remediation measures are often preferred (Hinds, 2023). Of these, biochar may increase soil adsorption of Pb through the high number of binding sites and other demobilising effects, such as increased pH (Guo et al., 2020). Current studies show that biochar largely reduces soil Pb human bioaccessibility. For example, Wang et al. (2022) reported significant decreases of 19.09–36.34% in Pb bioaccessibility with 1% water hyacinth-derived biochar in spiked silty loam topsoil. Through reducing the prevalence of mobile soil Pb, biochar may also consequently reduce plant uptake by up to 49–57% in lettuce (Medyńska-Juraszek et al., 2020; Vannini et al., 2021) and < 50% in root radish (Medyńska-Juraszek et al., 2020). Notably, biochar effects on root and bulb crop Pb uptake are less well documented.

Despite the moniker of ‘black gold’ and increasing interest in biochar research however, literature has begun to question the limitations of biochar and practical feasibility in the long-term (Tan & Yu, 2023). Notably, the remediation potential of biochar depends on biochar properties and interacting effects with local soil and plants (Medyńska-Juraszek et al., 2013). Soils with existing high soil organic matter (SOM) or clay content with a high number of binding sites, or soils with high pH and thus low Pb mobility potential, may then not benefit from the addition of biochar with similar properties. In urban agriculture however, the addition of plant crops may confound the interacting effects of soil and biochar.

As yet there is little study investigating the effects of biochar on Pb ingestion risk remediation in urban mono-crop agrisystems, particularly where biochar may have limited effect on soil properties. This research then systematically considers the effects of biochar on soil and vegetable Pb ingestion risk in four vegetable agrisystem soils with high binding potential (high SOM and clay) and alkaline pH. Observations of total vegetable and bioaccessible soil Pb concentrations are used to calculate health risk indices such as Average Daily Intake and Target Hazard Quotient (Gupta et al., 2022) to quantify independent and combined holistic urban grower and consumer ingestion risks. Combined soil and vegetable ingestion risk assessment is also not often considered, despite the common combined real-world soil and vegetable Pb exposure for urban growers, but is crucial for an accurate assessment of ingestion risk. This research will ultimately evaluate biochar potential for reducing urban agriculture Pb ingestion risks associated with the present study conditions. This will then underpin wider investigation and application of biochar in broader urban agricultural settings.

## Procedure

### Experimental design

A pot experiment was conducted in an outdoor 2 × 3 m polytunnel at Queen’s University Belfast, Northern Ireland, UK, between 28 February 2023 and 21 August 2023. Approximately 425 kg of soil was sourced from 22 locations at < 0.5 m depth across a local urban greenspace designated for future urban agriculture: Lower Botanic Gardens, Belfast, UK (54.578462: − 5.929747). This soil was identified as having high Pb content, high SOM, and high pH through an initial site investigation. Sampled soils were tested for Pb and composited into two conditions: ‘High lead’ (385.2 ± 202.4 mg/kg) and ‘Low lead’ (126.6 ± 39.90 mg/kg). Half of each High/Low Pb condition was amended with 5%w/w digestate biochar, locally sourced from AFBI, Northern Ireland (AFBI, 2025). Digestate biochar was selected for use in urban agrisystems as a local solution to removing phosphate-rich organic material (and its associated environmental issues) from agriculture/farmland agrisystems (Vienne et al., 2026). Soil and biochar properties are outlined in Table 1. Control and biochar soils were then split into four crop treatments with three replicates: No-crop, Lettuce, Carrot, and Garlic. These crops were chosen to represent leaf, root, and bulb edible parts. No-crop soils were tested prior to potting; only crops were grown in the polytunnel.

**Table 1:** Background soil and biochar properties

Background soil		Biochar	
Texture type	Clayey sand	Feedstock	Digestate
pH	8.31 ± 0.12	pH	11.9
		Bulk density, kg/m^3^	475
		Specific surface area, m^2^/g	463.68
		Ash content, %w/w	42.3
Organic matter, %w/w	8.42 ± 0.84		
Organic carbon, %w/w	6.38 ± 4.74	Organic carbon, %w/w	54.9
Nitrogen, %w/w	0.37 ± 0.13	Nitrogen, mg/kg	11,100
		Na_2_O, mg/kg	21,800
		MgO, mg/kg	28,200
		SiO_2_, mg/kg	158,000
		P_2_O_5_, mg/kg	40,600
		K_2_O, mg/kg	41,600
		CaO, mg/kg	69,900
		Fe_2_O_3_, mg/kg	11,700
As, mg/kg	15.54 ± 16.45	As, mg/kg	1.3
Cd, mg/kg	0.62 ± 0.46	Cd, mg/kg	< 0.2
Cr(III), mg/kg	38.89 ± 8.27	Cr(III), mg/kg	31
Cu, mg/kg	88.00 ± 54.31	Cu, mg/kg	163
Pb, mg/kg	222.3 ± 184.2	Pb, mg/kg	2
Ni, mg/kg	66.96 ± 21.65	Ni, mg/kg	16
Zn, mg/kg	208.3 ± 172.1	Zn, mg/kg	459

Biochar was sourced from the solid digestate fraction from an anaerobic digester at AFBI, Northern Ireland. Digestate pellets were pyrolysed at 675 ± 5 °C using a Biomacon C100–F Pyrolysis Boiler (R&S Biomass Equipment Ltd, Newtownstewart, NI). Laboratory characterisation of the biochar was undertaken by Eurofins Umwelt Gmbh, Germany (Table 1).

The 36 pots were arranged in a 6 × 6 fixed-order block design. Pots comprised large black 8L plant bags (0.22 m diameter × 0.22 m depth), with drainage holes, and individual drip trays. Pots were filled with 10 kg biochar-amended/control soil and then watered and set aside for 14 days to allow biochar activation. Seeds and bulbs were germinated indoors prior to the experiment. Pots were watered weekly after seeding. Average polytunnel temperature was 17.84 ± 5.55 °C and soil surface humidity was 82.36 ± 6.18%.

### Sample collection and preparation

Lettuce and post-lettuce soil was harvested on 22 May 2023, carrot and post-carrot soil was harvested on 27 July 2023, and garlic and post-garlic soil was harvested on 21 August 2023. Soil samples were oven-dried at 40 °C for 72 h (British Standards Institution, 2006) and hand-sieved to < 250 µm. Crop samples were harvested using stainless steel scissors. Non-edible parts were removed, and the remaining edible lettuce leaves, carrot root, and garlic cloves and 15 cm of garlic scape/stem were weighed, washed three times with tap water and rinsed with deionised water. Lettuce was oven-dried at 70 °C for 72 h. Carrot and garlic were oven-dried at 70 °C for 48 h. Dried vegetables were weighed and refined to a powder using a hand-blender.

### Soil total and bioaccessible concentrations

To determine soil total Pb, 0.02 g dried soil samples were digested using reverse aqua regia acid (9 mL HNO_3,_ 3 mL HCl) and microwave digestion (CEM Discover SPD Plus Microwave Digester), and subject to ICP-OES analysis. This protocol was deemed appropriate to determine accurate pseudo-total soil Pb concentrations (Caporale et al., 2023).

Soil Pb bioaccessibility was used as a proxy indicator of human health risk for each agrisystem and treatment, as within the Bioaccessibility (IVBA) → Relative Bioavailability (RBA) → Risk framework typically applied in risk assessment. This IVBA → RBA → Risk linkage assumes consistent IVBA-RBA correlations, soil homogeneity, and stable Pb speciation, which may not be applicable in some urban field conditions. However, bioaccessibility may considered a useful approximation of site-specific risk under these controlled study conditions, and with appropriate acknowledgement of uncertainty (see Supplementary Material). Bioaccessible soil Pb was determined using the Unified BARGE Bioaccessibility Method (Wragg et al., 2009). This method was designed for 0.6 g sample/60 mL fluid, though was adapted to 0.4 g/40 mL fluid for the present study. Digestive fluids were compiled as described in Wragg et al. (2009). Dried plant or soil (0.4 g) was added to a tube, mixed with 6 mL saliva fluid, and shaken for 10 s. Following this, 9 mL stomach fluid was added and tubes were subject to end-over-end rotation for 1 h at 37 °C to comprise the gastric phase. Finally, 18 mL duodenal fluid and 6 mL bile fluid were added and tubes were rotated for 4 h at 37 °C to comprise the gastrointestinal phase. Digested samples were centrifuged, and supernatants were filtered through a 0.45um syringe filter before ICP-MS analysis (Thermo Scientific iCAPQc ICP-MS).

Each batch of 10 samples comprised one blank sample, one BGS102 reference sample, and two duplicates. Average reference sample recovery rates were 86.9% in the gastric phase. Duplicate samples reported 3.68% relative standard deviation in the gastric phase. Blank Unified BARGE Bioaccessibility Method (UBM) samples presented an average 2.23% of sample concentrations.

Bioaccessible fractions (Pb_BAF_ %) were calculated using the total (Pb_T_) and bioaccessible (Pb_BAC_) Pb concentrations (mg/kg) as follows.

1 $${Pb}_{BAF}= \text{ } \frac{{Pb}_{BAC}}{{Pb}_{T}} \, \times 100\mathrm{\%}$$

#### Crop total concentrations and bioconcentration factors (BCF)

For each plant sample, 100 mg plant matter and 2 mL pure 69% HNO_3_ were added to labelled 50 mL polycarbonate tubes. After 24 h, 2 mL H_2_O_2_ was added and samples were subject to microwave digestion at 95 °C for 30 min, with a 30-min cooldown. After digestion, samples were diluted to 30 mL with deionised water and 15µL Rh was added as an internal standard. Extractions were subject to ICP-MS analysis (Thermo Scientific iCAP TQ ICP-MS).

Three blank samples, and three certified reference samples (Certified-NCS ZC73020-Garlic) were included in analysis. The three blank samples reported an average 0.032 ± 0.003 mg/kg. Average reference sample recovery rates were 78.7%.

The Bioconcentration Factor (BCF) was calculated using the total soil (Pb_soil_) and total vegetable (Pb_veg_) Pb concentration (mg/kg) as follows. A BCF > 1 indicates a hyperaccumulator (Akhter et al., 2022).

2 $$BCF= \text{ } \frac{{Pb}_{veg}}{{Pb}_{soil}}$$

#### Risk indices

Risk indices were applied to 1) soil bioaccessible concentrations to indicate soil ingestion risk for adult urban growers; 2) total vegetable concentrations to indicate vegetable consumption risks for adult urban consumers; and 3) combined soil bioaccessible and vegetable total concentrations to indicate Pb ingestion risks for adults that both grow and consume urban crops. The combined assessment of soil and vegetable risk was deemed a real-world assessment of risk to urban residents that both grow and consume vegetables on the same site.

Average daily intake

The average daily intake (ADI) quantifies daily ingestion in mg/kg/day, based on the USEPA method reported in Billmann et al. (2023).

3 $${ADI}_{soil}= \text{ } \left(\frac{{Pb}_{soil} \, \times (BAF \, \times 0.8) \, \times EF \, \times ED \, \times IR}{BW \, \times AT}\right) \, \times \, {10}^{-6}$$

4 $${ADI}_{veg}= \text{ } \left(\frac{{Pb}_{veg}\times C \, \times EF \, \times ED \, \times IR}{BW \, \times AT}\right) \, \times \, {10}^{-3}$$

Where Pb_soil/veg_ = Total Pb concentration in soil or crop (dry weight mg/kg); BAF = Bioaccessible Fraction (%); (BAFx0.8) = regression model conversion of BAF to Relative Bioavailability (RBA) (Cocerva et al., 2024); C = vegetable dry-to-fresh weight conversion (0.096 for lettuce, and 0.103 for carrot and garlic) (Environment Agency, 2009); EF = Exposure frequency (365 days); ED = Exposure duration (75 years) (Environment Agency, 2009); IR = daily soil ingestion rate for growers/gardeners (50 mg/day soil, 161 g/day crop) (Hinks et al., 2016; Jeffries & Martin, 2009); BW = Body weight of average 16 < 65 y adult female human (70 kg) (Environment Agency, 2009); AT = average exposure time (EFxED).

Target hazard quotient (non-carcinogenic risk)

The Target Hazard Quotient (THQ) quantifies aggregated non-carcinogenic risks linked to regular consumption of contaminated soils and plants. A THQ of > 1 indicates risk of metal toxicity (Gupta et al., 2022).

5 $$THQ= \text{ } \frac{ADI}{RfD}$$

Where ADI = described above; RfD = oral reference consumption dose of lead (0.0035 mg/kg/day) (Mehta et al., 2020).

Cancer slope risk (carcinogenic risk)

The Cancer slope Risk (CR) estimates the upper-bound probability that an individual will develop cancer if exposed to soil and vegetables over 70 years. CR > 0.0001 suggests an unacceptable health risk; CR 0.0001 > 0.000001 poses moderate acceptable risk, and CR < 0.000001 poses a negligible threat (Nag & Cummins, 2022).

6 $$CR= \text{ } ADI \, \times CSF$$

Where ADI = defined above; CSF = oral cancer slope factor for lead (0.0085 mg/kg/day) (Nag & Cummins, 2022).

### Statistical analysis

Statistical analysis was performed using RStudio (2022). All data were checked for normal distribution (Shapiro Wilk test) and homogeneity of variance (Levene’s test). Two-way analysis of variance (ANOVA) and post-hoc Tukey tests were applied where data met test assumptions. Soil attributes were also compared using Principal Component Analysis (PCA) in RStudio (2022). Data were normalised by scaling to unit variance (subtracting the mean and dividing by the standard deviation for all data points) before analysis. Percentage change between treatments was calculated as follows:

7 $$\frac{A-B}{B} \, \times 100\mathrm{\%}$$

Where A = Value after treatment, B = Value before treatment.

### Uncertainty and sensitivity analysis

Human health risk assessment encompasses a range of values for common variables that may be applied under different risk scenarios. As a result, derived risk assessment values inherently possess some level of uncertainty. Monte Carlo simulations utilise repeated random measures of applied risk parameter ranges, rather than single point risk parameter values, to yield the frequency/probability distribution of exposure variables beyond the standard deviation and percentile measures (Fallahizadeh et al., 2025; Mianeh et al., 2025). This then increases the model robustness and representativeness for risk analysis (Moazamnia et al., 2024).

Adult exposure risk was explored in the present study. Site-specific soil and vegetable concentration (and soil BAF) ranges were applied in the simulations (Supplementary Material, Table 1, Table 2). In addition, Exposure Frequency (EF), Ingestion Rate (IR), and Body Weight (BW) ranges for adults were also considered in the simulations (Supplementary Material, Table 1, 2). Microsoft Excel-based add-in Argo was used to compile simulations and sensitivity analyses. Simulations were run using continuous values within each parameter range. Simulation sets were undertaken for non-carcinogenic risks (THQ) for each treatment combination in the study (High or low Pb content/Vegetable agrisystem/biochar amendment). In total, 280,000 random repeat simulations were run, split into 10,000 simulations per 16 soil ingestion risk treatment combinations and 12 vegetable combinations. A frequency-probability histogram was produced in Microsoft Excel to show the distribution of values procured using the range of parameter inputs.

**Table 2:** Soil pH and organic matter differences between control and biochar treatments across agrisystems. HNB = high Pb, no biochar; HB = high Pb, biochar; LNB = low Pb, no biochar; LB = low Pb, biochar. Bold text indicates significant differences between control and biochar treatments

	No-crop				Lettuce				Carrot				Garlic			
	HNB	HB	LNB	LB	HNB	HB	LNB	LB	HNB	HB	LNB	LB	HNB	HB	LNB	LB
pH	8.38 ± 0.07	8.33 ± 0.04	**8.14 ± 0.07**	**8.38 ± 0.10**	**7.99 ± 0.12**	**8.70 ± 0.17**	**7.93 ± 0.03**	**8.89 ± 0.13**	**8.01 ± 0.05**	**8.80 ± 0.04**	**7.93 ± 0.02**	**8.92 ± 0.06**	**8.16 ± 0.03**	**8.92 ± 0.06**	**8.07 ± 0.07**	**8.93 ± 0.07**
SOM / %	8.34 ± 0.90	8.32 ± 1.40	8.19 ± 0.54	8.83 ± 0.81	**9.07 ± 0.29**	**10.02 ± 0.26**	**8.12 ± 0.27**	**10.20 ± 0.20**	**7.85 ± 0.26**	**9.15 ± 0.76**	**7.79 ± 0.14**	**8.62 ± 0.25**	8.49 ± 0.34	9.39 ± 1.68	**7.57 ± 0.39**	**9.50 ± 0.19**

Subsequent sensitivity analyses were undertaken to determine the contributing variables to the THQ for each treatment (Mianeh et al., 2025). Analyses and tornado charts were compiled using Microsoft Excel Argo add-in software using the 10,000 repeated simulations in the Monte Carlo analysis.

## Results and discussion

### Soil pH and SOM

Table 2 describes the impact of biochar on soil pH and organic matter. Notably, soil pH was increased with biochar application in most agrisystems, with the exception of high Pb no-crop agrisystems, though all soils remain classified alkaline. This alkalinisation was also observed in studies investigating acidic soils (Liu et al., 2025; Lu et al., 2025) and was attributed to the high inherent pH in biochar. Soil organic matter was again increased in many agrisystems after biochar addition (Table 2), again attributed to the inherent organic feedstock of biochar (Liu et al., 2025; Lu et al., 2025).

### Soil total Pb

**Fig. 1:**
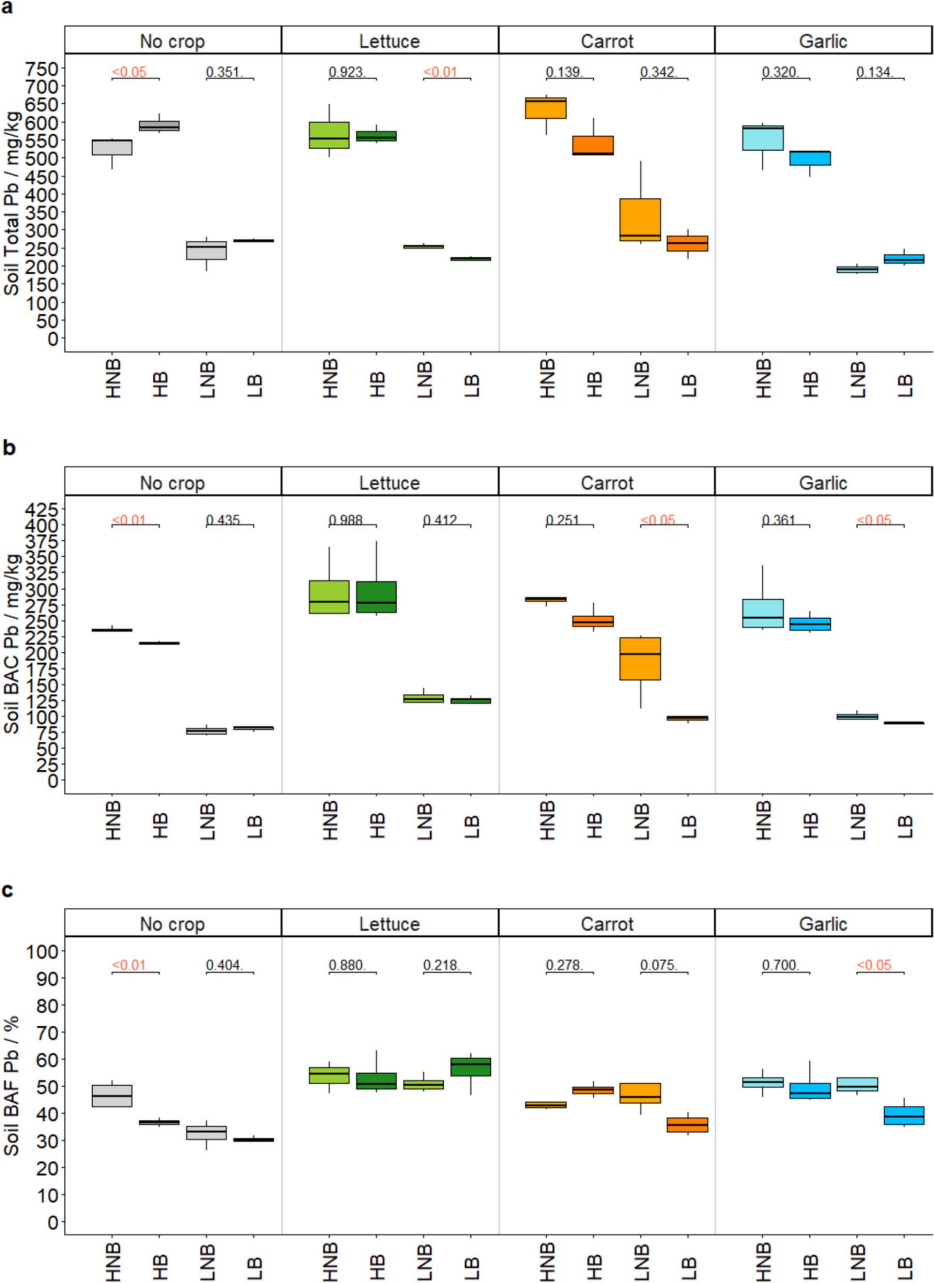
Total soil Pb across vegetable agrisystems (mg/kg) (**a**) and bioaccessible soil Pb concentration (BAC) (mg/kg) (**b**) and bioaccessible soil Pb fraction (BAF) (%) (**c**). HNB = high Pb, no biochar; HB = high Pb, biochar; LNB = low Pb, no biochar; LB = low Pb, biochar. ANOVA p-values are reported for comparisons between control and biochar (red colour indicates significant values)

Average total soil Pb was 557.2 ± 60.22 mg/kg for high Pb conditions, and 250.2 ± 61.29 mg/kg for low Pb conditions. All soil total Pb concentrations were greater than the UK C4SL guideline values used to identify contaminated land, of 80 mg/kg for allotments (Harries et al., 2014) (Fig. 1a). Generally, the range of reported values in high Pb conditions was greater than for low Pb conditions. This may be attributed to sparse and variable distribution of anthropogenic Pb in high Pb conditions, compared to lower levels.

Broadly, biochar-amended soil presented marginally lower total Pb (395.3 ± 160.7 mg/kg) than control soil (412.1 ± 174.8 mg/kg). In assessing effects within agrisystems however, biochar typically increased total soil Pb in No-crop (unplanted) agrisystems and reduced total soil Pb in planted agrisystems, to varying degrees of significance (Fig. 1a).

Total soil Pb is often observed to decrease after biochar addition, due to its adsorptive properties (Khan et al., 2020a, 2020b, 2020c), and also due to natural soil PTE dilution with ‘clean’ biochar (Qasim et al., 2021). This effect was expected to be most pronounced in no-crop systems due to the lack of confounding influences from vegetable acidic root exudates and altered soil biome. In unplanted systems, Zhang et al. (2021) and Gomes et al. (2022) comparably reported decreases of ~ 45% and ~ 11% total Pb with < 20% lime-zeolite-biochar and 10% sugarcane biochar, respectively. In the present study, biochar alternately increased total Pb in the high and low Pb no-crop agrisystems. This effect was not attributed to biochar Pb content, as the study biochar contained only 2 mg/kg Pb that would have instead created a diluting effect. Instead, the extraction method may preferentially release anthropogenic biochar-bound soil Pb in the higher Pb conditions, compared to the recalcitrant geogenic Pb assumed in low Pb conditions. Differing extraction methods may also explain differing results between literature and the present study; for example, Zhang et al., (2021) utilised CaCl_2_ extractions to yield significant total Pb decreases after biochar application, compared to reverse aqua-regia microwave digestion in the present study.

For planted systems, the present study reported statistically-significant decreases in total soil Pb in the low Pb lettuce agrisystem (−12.8%), compared to low Pb control treatments. This is somewhat supported in literature by the non-significant effects of 5%w/w black silt biochar on soil total Pb in lettuce agrisystems reported by Vannini et al. (2021), and by Lian et al. (2023) who reported no significant effects with pig manure biochar under cabbage systems.

### Soil bioaccessible Pb

As observed in literature, gastric phase Pb bioaccessibility was significantly higher than gastrointestinal bioaccessibility (Du et al., 2020; Wragg & Cave, 2003). Thus gastric values were used to represent worst-case scenario risk analysis (Wragg & Cave, 2003).

Soil Pb bioaccessible concentrations (BACs) were significantly higher in high Pb conditions (260.9 ± 39.78 mg/kg) compared to low (109.6 ± 37.50 mg/kg), though BAFs were not significantly different (47.80 ± 6.79% high Pb; 43.16 ± 10.62% low Pb). As per total Pb, the range of reported BAC in high Pb conditions was greater than for low Pb conditions, possibly linked to variation in Pb source. Typically, BAFs were observed to be between 28.03–66.36%, which is consistent with assumptions used in UK contaminated land risk assessments (Harries et al., 2014) (Fig. 1b and c).

Biochar-amended soil presented marginally lower bioaccessible Pb (174.2 ± 84.21 mg/kg; 43.59 ± 9.90%) than control soil (196.3 ± 86.41 mg/kg; 47.36 ± 8.44%). Comparably, Yang et al. (2016) reported a reduction in bioaccessibility of 9.8% in bagasse biochar-mended soils under cabbage mustard seedlings at 640.5 mg/kg Pb soil concentrations, compared to reductions of 3.8% in digestate biochar-amended at similar 547.9 mg/kg (high) Pb soil concentrations in the present study. Biochar reduces Pb mobility and encourages complexation by increasing soil pH and CEC and increasing SOM binding sites (Albert et al., 2021). There is far greater potential for alkaline biochar to alter acidic soil chemical conditions, resulting in increased complexation effects and more significant reductions in mobility (Liu et al., 2025; Lu et al., 2025), as seen in soils of pH 5.8 in Yang et al., (2016). However, the present study soil was clayey sand with higher SOM (8.42 ± 0.84%) and higher pH (8.31 ± 0.12). As observed in the present study, adding alkaline biochar to alkaline soil with higher SOM results in very limited chemical change and complexation potential compared to studies in acidic, low-SOM soils, such as Yang et al. (2016). In addition, digestate biochar complexation potential may be limited due to its inherent complex structure and high cation and anion loading (Table 1). The limited effects of biochar may also be attributed to the lower application rate used in the present study (5%w/w) compared to other literature (> 10%; Gomes et al., 2022; Park et al., 2011). It may also be attributed to the use of the Unified BARGE Bioaccessibility Method (UBM). Though this assay often reports higher bioaccessible concentrations than other assays (Zheng et al., 2022), it may still not be comparable to single-phase acid extractions used to represent bioavailability that are capable of greater Pb extraction, such as those used in Park et al. (2011) and Vannini et al. (2021).

Where PTE speciation distribution is the same across samples, bioaccessible concentrations are often observed as proportional to total concentrations (Li et al., 2024; Netherway et al., 2019). Here however, biochar effects on bioaccessibility in specific agrisystems did not always correlate to effects seen for total Pb. For example, high Pb no-crop BACs significantly decreased with biochar whereas total Pb was significantly increased in the same agrisystem. Bioaccessible Pb in low Pb carrot and garlic agrisystems were also significantly decreased where only low Pb lettuce total Pb was decreased. Ultimately, this highlights the need to incorporate site/agrisystem-specific bioaccessible concentrations in risk assessment.

Between agrisystems, biochar generally had a greater effect in high Pb soils for the unplanted no-crop systems, and in low Pb agrisystems for planted agrisystems (Fig. 1b and c). Soil BAC/BAF differences between agrisystems may be attributed to the complex interactions between soil, plants and biochar. Lettuce, carrot, and garlic were chosen for their different leaf cover (and thus detritus and soil shading), root distribution, chemical root exudate composition (Poggio et al., 2009; Weaver & Bruner, 1927). Biochar is known to improve plant growth/yield and thus increase many of these attributes (Knoblauch et al., 2021), consequently confounding biochar effects across different agrisystem soils. Greater biochar effects in planted carrot and garlic agrisystem soil may be attributed to a longer crop growth period and thus longer plant-soil-biochar interactions, compared to short-term lettuce interactions. Janus et al., (2018) reported no change in Pb bioaccessibility in Miscanthus and hardwood biochar-amended soils until up to 6 months after application. Peak biochar activation period in the present study then aligned with the ‘longer-term’ carrot and garlic and had lesser effect on the ‘shorter-term’ lettuce. No-crop systems did not have the confounding effects and altered soil microbiomes due to planted root systems to alter biochar effectiveness. This does not explain no effects in high Pb planted systems, however. In this case, the higher concentrations may inhibit this activity and limit biochar effects.

### Crop total Pb

**Fig. 2:**
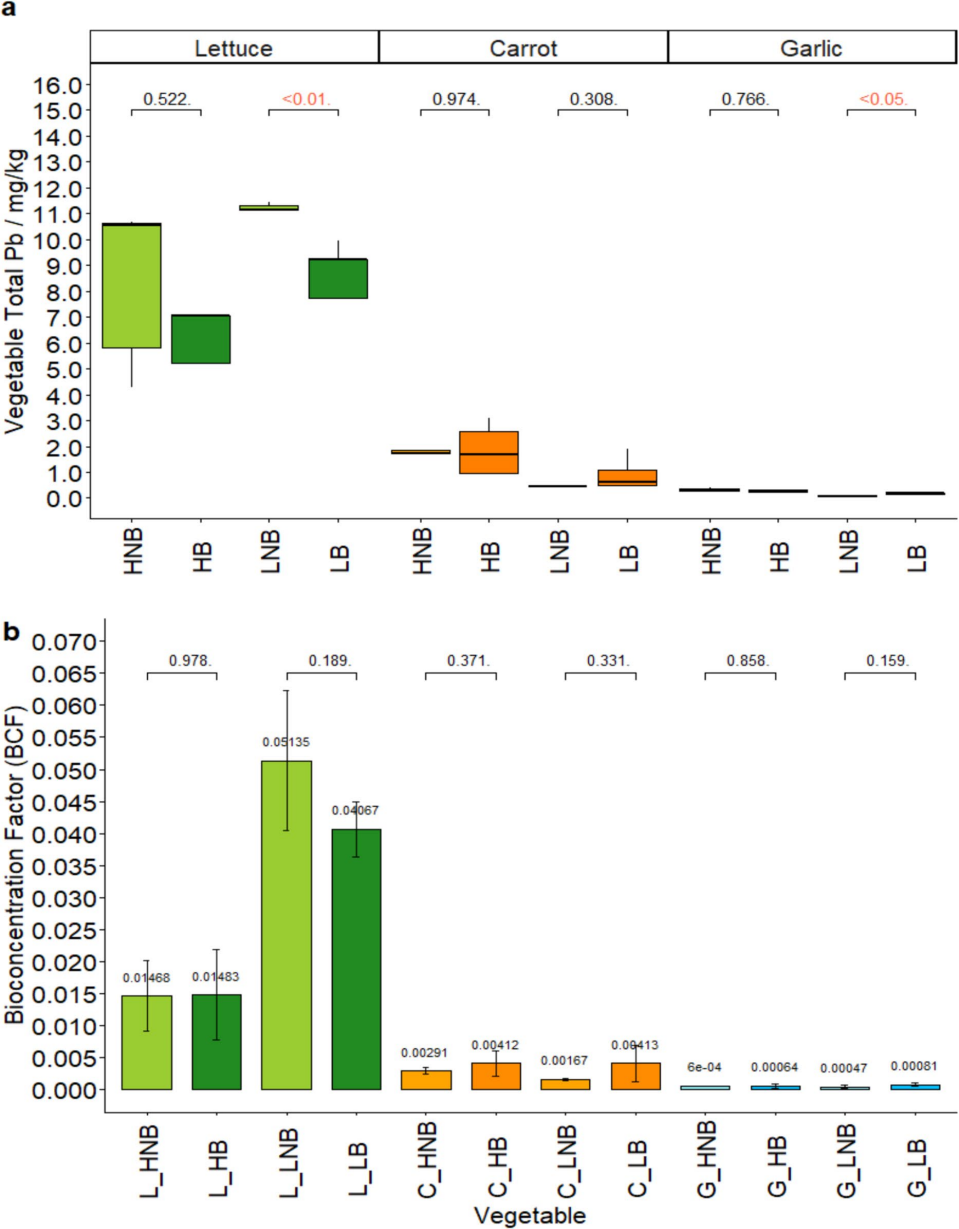
**(a)** Total vegetable Pb across agrisystems (mg/kg). **(b)** Bioconcentration Factors (BCF) for vegetable agrisystems. HNB = high Pb, no biochar; HB = high Pb, biochar; LNB = low Pb, no biochar; LB = low Pb, biochar. ANOVA p-values are reported for comparisons between control and biochar (red colour indicates significant values)

Only garlic from control treatments reported average total Pb concentrations below the maximum concentration international food standards: 0.3 mg/kg for leaf vegetables and 0.1 mg/kg for root/bulb vegetables (FAO, 2023) (Fig. 2a). All other vegetables exceeded the maximum permitted Pb concentration.

There was no significant difference in vegetable total Pb between high (3.58 ± 4.05 mg/kg) and low (4.18 ± 5.51 mg/kg) Pb condition soils (Fig. 2a). Vegetable Pb was most strongly correlated to vegetable species. Notably, lettuce contained much higher Pb (9.72 ± 3.38 mg/kg), compared to carrot (1.47 ± 0.90 mg/kg) and garlic (0.23 ± 0.12 mg/kg) (Fig. 2a) (*p* < 0.01). Guerrieri et al., (2024) and Pavlíková et al., (2023) similarly report that leaf vegetables accumulate greater Pb concentrations compared to root and bulb vegetables. Pb accumulation in lettuce may be linked to soil water and rapid water cycling in this short-term crop, compared to subterranean vegetables, which may increase uptake of Pb mobilised in soil water (Kim et al., 2019). As a high value-high yield crop often grown in the local area, this has exposure implications for growers.

Comparing biochar effects within High/Low Pb vegetable agrisystems; biochar significantly reduced average lettuce Pb concentrations in low Pb soils by 31% (from 13.10 mg/kg control to 8.97 mg/kg biochar, *p* < 0.01), and significantly increased average garlic concentrations in low Pb soils by 50% (from 0.09 mg/kg control to 0.18 mg/kg biochar, *p* < 0.05) (Fig. 2a). Similar leaf vegetable Pb reductions are observed in literature, for example: 33% reduction in lettuce Pb with 5%w/w wheat straw biochar (Medyńska-Juraszek et al., 2013); 33.54% reduction in Chinese cabbage with 5% rice-straw biochar (Salam et al., 2019); and 36.2% reduction in cilantro and 50.5% reduction in spinach using 3%w/w hardwood biochar (A. Z. Khan, Ding, et al., 2020). Significant increases in garlic Pb after biochar addition may not be representative as the observed concentrations in garlic were very low, as such, a small increase of 0.09 mg/kg resulted in a significant result. In contrast, Pb accumulation in control/biochar treatments lettuce with higher concentrations did not yield a significant difference, despite a greater average change (8.48 mg/kg to 7.45 mg/kg with biochar). The small average 0.09 mg/kg increase in Pb garlic after biochar addition may be attributed to differences in soil Pb species and soil pH/SOM/CEC conditions within individual treatment plots, rather than the effects of biochar. No significant effects of biochar were observed in other agrisystems, though this may be masked by very low concentrations (Fig. 2a). Little biochar effect was observed in carrot and garlic in the present study. In contrast, Hegab et al. (2016) and Cheng et al. (2020) reported significant Pb reductions in carrots and radish, after amending soils with 5% rice straw biochar and 3%w/w rice-husk biochar, respectively. Differences may be attributed to the greater change in soil properties after biochar addition in Hegab et al. (2016) (from neutral pH, low SOM, and loamy sand soil) and to the additional use of metal-immobilizing bacteria in Cheng et al. (2020).

In assessing bioconcentration factors (BCFs), lettuce accumulated far greater Pb than carrot or vegetables (Fig. 2b). Extensive aerial deposition and dust disturbance during irrigation may influence lettuce Pb. Uzu et al., (2010) reported that 98.5% of leaf Pb may come from aerial deposition compared to root uptake, due to easy entry through stomata and low fibre tissues. Conversely, subterranean carrot and garlic edible matter may possess stronger defence mechanisms and fibrous structures against long-term soil exposure, thus edible root and bulb structures may present lower Pb (Pandey et al., 2025). Lettuce and garlic also accumulated greater Pb concentrations in the low Pb conditions (B). This may be attributed to the differential source, species, and consequent complexation potential of Pb in the high and low conditions.

Biochar had no statistically-significant effects on plant Pb accumulation, though there was a minimal broad trend showing increased accumulation in some carrot and garlic samples, with variable effects on lettuce (Fig. 2b).This, and similar trends in vegetable concentrations, suggests that biochar may either mobilise soil Pb (not confirmed by soil total/bioaccessible Pb) or, perhaps more likely, improve plant shoot and root growth as reported in Zou et al., (2021), and thus support more prevalent Pb uptake through water channels and higher PTE tolerance.

### Soil and plant PCA

**Fig. 3:**
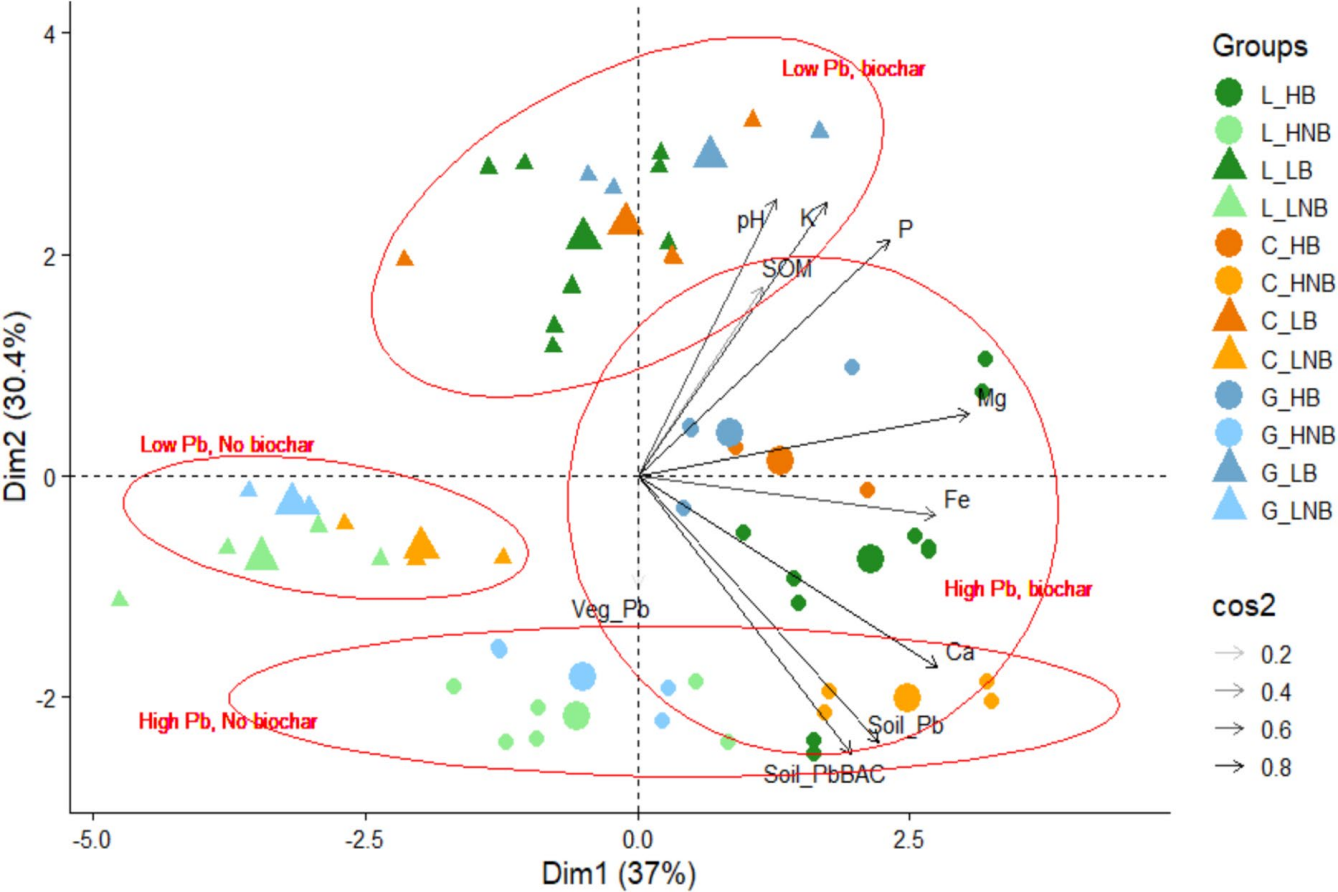
PCA comparing soil total and bioaccessible Pb, vegetable total Pb, and soil parameters. L_ = Lettuce, C_ = Carrot, G_ = Garlic; _ HNB = high Pb, no biochar; _HB = high Pb, biochar; _LNB = low Pb, no biochar; _LB = low Pb, biochar. The length of the arrows and size of the points depict the quality of the relationship between variables (cos2). Red circle annotations indicate 95% confidence levels for labelled grouped variables using a multivariate distribution

Dim 1 and Dim 2 (Fig. 3) describe 67.4% variance between variables (Dim 1: 37.040%; Dim 2: 30.355%; Dim 3: 13.365%; Dim 4: 9.545%). This pattern illustrates the weaker link between vegetable total Pb and soil parameters, particularly for biochar. McBride et al., (2014) similarly reported low PTE transfer likely due to soil characteristics affecting phytoavailability, which, in this case, would be further confounded by biochar application. Dim 1 showed clear distinctions between high and low total soil Pb concentrations, notably between high Pb-biochar; and low Pb-no biochar treatments (see annotations, Fig. 3). Dim 1 was also strongly controlled by Fe and Mg, which is consistent with the known co-variation of these elements with Pb in contaminated soils (Alloway, 2013). Dim 2 related to control/biochar treatments, which was likely driven by increases in soil pH and SOM typically associated with biochar application (Lehmann & Joseph, 2015). Broadly, soil pH, K, SOM and P (related to biochar), and Ca, total and BAC Pb (related to soil high/low Pb conditions), affected both dimensions.

### Risk indices

**Fig. 4:**
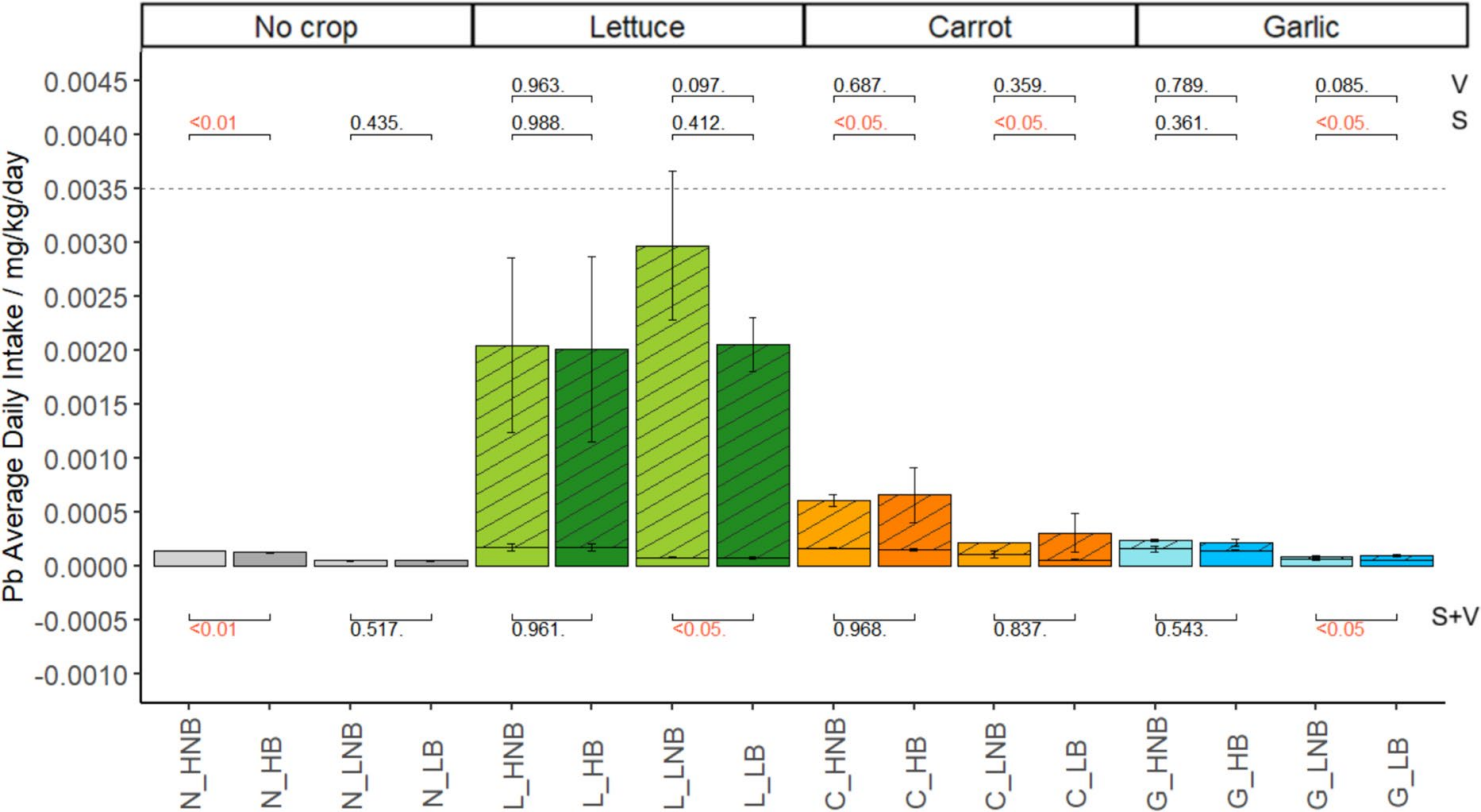
Stacked vegetable (patterned) and soil (plain) Average Daily Intake (ADI). ANOVA p-values compare control/biochar for soil (S), vegetable (V), and combined soil + vegetable (S + V); red values indicate significance. HNB = high Pb, no biochar; HB = high Pb, biochar; LNB = low Pb, no biochar; LB = low Pb, biochar. Dashed line indicates oral reference dose 0.0035 mg/kg/day (Gupta et al., 2022)

All soil and vegetable ADI values were lower than the Pb USEPA oral reference dose value (RfD) of 0.0035 mg/kg/day (Gupta et al., 2022; Shaheen et al., 2016) (Fig. 4). All soil THQs were lower than the THQ threshold of 1, and a single vegetable THQ exceeded the threshold (Low Pb/lettuce/control; 1.053). All soil and vegetable CRs were lower than the significant risk threshold of 0.0001.

Different ingestion risks were observed for different types of exposure. Table 3, 4, 5 describe the agrisystem Average Daily Intake (ADI), Target Hazard Quotient (THQ) and Cancer Risk (CR) for soil-only, vegetable-only, and combined soil and vegetable Pb exposure, respectively. Overall, greater daily Pb intake was associated with soils in garlic and no-crop agrisystems, and with vegetables in lettuce and carrot agrisystems.

**Table 3:** Soil-only average daily intake (ADI), Target hazard quotient (THQ) and Cancer risk (CR) in agrisystems. Percent change from control to biochar treatments, and p-values are described. Significant values reported in bold text.

Agrisystem	**Treatment**	**ADI**	**THQ**	**CR**	**% change**	**p-value**
No crop	HNB	1.34 E-04	3.80 E-02	1.14 E-06	** − 9.3**	** < 0.01**
	HB	1.22 E-04	3.48 E-02	1.04 E-06		
	LNB	4.41 E-05	1.26 E-02	3.75 E-07	+ 4.8	0.435
	LB	4.62 E-05	1.32 E-02	3.93 E-07		
Lettuce	HNB	1.69 E-04	4.83 E-02	1.44 E-06	+ 0.2	0.988
	HB	1.69 E-04	4.84 E-02	1.44 E-06		
	LNB	7.36 E-05	2.11 E-02	6.28 E-07	− 5.7	0.412
	LB	6.97 E-05	1.99 E-02	5.93 E-07		
Carrot	HNB	1.61 E-04	4.59 E-02	1.37 E-06	** − 10.9**	** < 0.05**
	HB	1.43 E-04	4.09 E-02	1.22 E-06		
	LNB	1.04 E-04	2.98 E-02	8.86 E-07	** − 47.4**	** < 0.05**
	LB	5.49 E-05	1.57 E-02	4.66 E-07		
Garlic	HNB	1.54 E-04	4.40 E-02	1.31 E-06	− 8.9	0.361
	HB	1.4 E-04	4.01 E-02	1.19 E-06		
	LNB	5.72 E-05	1.63 E-02	4.86 E-07	** − 11.1**	** < 0.05**
	LB	5.08 E-05	1.45 E-02	4.32 E-07

**Table 4:** Vegetable-only Average Daily Intake (ADI), Target Hazard Quotient (THQ) and Cancer Risk (CR) in agrisystems. Percent change from control to biochar treatments, and p-values are described. Significant values reported in bold text.

Agrisystem	**Treatment**	**ADI**	**THQ**	**CR**	**% change**	**p-value**
Lettuce	HNB	1.87 E-03	5.35 E-01	1.59 E-05	− 1.9	0.963
	HB	1.84 E-03	5.26 E-01	1.56 E-05		
	LNB	2.89 E-03	8.27 E-01	2.46 E-05	− 31.7	0.097
	LB	1.98 E-03	5.66 E-01	1.68 E-05		
Carrot	HNB	4.44 E-04	1.27 E-01	3.77 E-06	+ 14.9	0.687
	HB	5.10 E-04	1.46 E-01	4.33 E-06		
	LNB	1.07 E-04	3.05 E-02	9.09 E-07	+ 133.2	0.359
	LB	2.50 E-04	7.13 E-02	2.12 E-06		
Garlic	HNB	7.83 E-05	2.24 E-02	6.65 E-07	− 7.2	0.789
	HB	7.26 E-05	2.07 E-02	6.17 E-07		
	LNB	2.09 E-05	5.98 E-03	1.78 E-07	+ 101.7	0.085
	LB	4.23 E-05	1.12 E-02	3.59 E-07

**Table 5:** Combined soil and vegetable average daily intake (ADI), Target hazard quotient (THQ) and Cancer risk (CR) in agrisystems. Percent change from control to biochar treatments, and p-values are described. Significant values reported in bold text.

Agrisystem	Treatment	ADI	THQ	CR	% change	p-value
No crop	HNB	1.34 E-04	3.80 E-02	1.14 E-06	** − 9.3**	** < 0.01**
	HB	1.22 E-04	3.48 E-02	1.04 E-06		
	LNB	4.41 E-05	1.26 E-02	3.75 E-07	+ 4.8	0.517
	LB	4.62 E-05	1.32 E-02	3.93 E-07		
Lettuce	HNB	2.04 E-03	5.83 E-01	1.74 E-05	− 1.7	0.961
	HB	2.01 E-03	5.74 E-01	1.71 E-05		
	LNB	2.97 E-03	8.48 E-01	2.52 E-05	** − 44.8**	** < 0.05**
	LB	2.05 E-03	5.86 E-01	1.74 E-05		
Carrot	HNB	6.04 E-04	1.73 E-01	5.14 E-06	+ 7.4	0.968
	HB	6.53 E-04	1.87 E-01	5.55 E-06		
	LNB	2.11 E-04	6.03 E-02	1.79 E-06	+ 30.6	0.837
	LB	3.04 E-04	8.70 E-02	2.59 E-06		
Garlic	HNB	2.32 E-04	6.64 E-02	1.97 E-06	− 9.1	0.543
	HB	2.13 E-04	6.08 E-02	1.81 E-06		
	LNB	7.81 E-05	2.23 E-02	6.64 E-07	** + 16.1**	** < 0.05**
	LB	9.31 E-05	2.66 E-02	7.91 E-07		

Broadly, biochar had less effect on risk indices than anticipated. The variable effects of biochar on soil and vegetable ADI, THQ, and CR between agrisystems can be attributed to those identified for soil bioaccessible Pb and total vegetable Pb, respectively, as this was the singular independent variable in the risk indices equations. However, trends in biochar efficacy across agrisystems differed slightly between bioaccessible soil/total vegetable Pb and the derived risk indices. This was likely due to the minimal measured effects of biochar in each agrisystem that were differentially amplified in the calculation. Ultimately, site-specific assessment and site-tailored application rates would need to be considered for optimal risk assessment.

#### Soil-only risk indices

Soil ingestion risks were greater than vegetable ingestion risks in garlic (and no-crop) agrisystems, though the opposite was true to carrot and lettuce agrisystems.

For soil ingestion (Table 3), there was a general ADI, THQ, and CR hierarchy of Lettuce soil > Carrot soil > Garlic soil > No-crop (unplanted) soil for each high/low Pb, control/biochar treatment combination. For example, in High Pb/control reported THQ: 4.83 E-02 (lettuce) > 4.59 E-02 (carrot) > 4.40 E-02 (garlic) > 3.80 E-02 (no-crop) (Table 3). Soil ingestion risks were then greater in planted agrisystem soil compared to no-crop soil.

Comparably, for no-crop systems, Zhao et al. (2022) reported a similar soil Pb THQ of 0.034–0.784 in a no-crop urban brownfield site in China, despite using different THQ calculation parameters. Ene et al. (2024) reported higher soil THQs of 0.0585–0.395 from no-crop urban parks in Romania. These greater THQs may again be due to the THQ calculation parameters as Ene et al. (2024) based the equation on children where anticipated pica-related ingestion and proportional biological effects, and thus THQ risk, would be greater. In planted systems, Kicińska et al., (2024) similarly reported high adult soil Pb THQs of < 0.4 in rural-agricultural lettuce agrisystem soils across Poland. As risk indices are based on soil total / bioaccessible Pb concentrations, this hierarchy may be explained by the differential effects of crops on respective soil Pb content as discussed in Sect. "Soil total Pb" and "Soil bioaccessible Pb".

Biochar had the most significant risk reduction effects for growers with soil-only exposure. Biochar significantly reduced average soil-only ADI, THQ and CR in high Pb no-crop, high Pb carrot, low Pb carrot, and in low Pb garlic agrisystems, with no effects in other agrisystems. This indicates that biochar effects are greater for longer-term crops. This coincides with the activation period of biochar, with peak effects coinciding with carrot and garlic harvesting after 6 months, as also observed in Janus et al., (2018).

#### Vegetable-only risk indices

For vegetable ingestion (Table 4), the ADI/THQ/CR hierarchy similarly reported Lettuce > Carrot > Garlic. Vegetables reported greater Pb ingestion risk than soils in lettuce and carrot agrisystems.

There were no significant differences between vegetable control and biochar agrisystems, which suggests that biochar does not affect daily Pb intake values from vegetables. This differs to the significant effects of biochar observed in total vegetable Pb in low Pb lettuce and garlic. Vegetable THQ are variable across literature, ranging from high < 15 for lettuce (Habte et al., 2023) to low 0.003 for carrot (Shaheen et al., 2016), however the present study vegetable THQs are generally comparable to those reported for leaf (Baghaie & Fereydoni, 2019), root (Nowar et al., 2024), and bulb (Nowar et al., 2024).

The minimal and variable effects of biochar on vegetable risk indices observed in the present study are supported in literature. Nawab et al. (2018) reported a non-significant 39.0% ADI reduction in pea using 2%w/w non-specified biochar, which is similar to the 31.7% ADI decrease with 5%w/w digestate biochar in low Pb lettuce in the present study. S. Zhang et al. (2023) similarly reported minimal effects of 5%w/w sludge biochar on Pak Choi Pb THQ (0.0433–0.0711). A. Z. Khan et al., (2020a, 2020b, 2020c) reported high THQ of 2.28 in cilantro and spinach from control plots, though significant reductions to 1.61 with 3%w/w poplar biochar, and to 1.26 with 3%w/w sugarcane biochar. This was attributed to the reduction of mobile Pb soil and thus vegetable uptake. The present study did not report strong correlations between local soil and vegetable Pb however, which may explain the non-significant effect of biochar on vegetable Pb concentration-dependent ADI and THQ. The limited effects of biochar on soil properties and Pb in the present study may then influence the minimal effects observed in vegetables.

As vegetable ingestion dominated Pb risk exposure in the lettuce and carrot agrisystems, vegetable bioaccessibility may be useful to determine accurate risk assessment in future studies. There is increasing literature exploring vegetable bioaccessibility, though current findings are somewhat variable (Newell et al., 2025).

#### Combined soil and vegetable risk indices

Comparing the effects of biochar on combined soil and vegetable Pb ingestion risks can determine biochar effects on the major ingestion risk factors (soil or vegetable) for urban growers for each mono-crop agrisystem. This then gives a holistic impression of the effects of biochar, compared to individual effects of singular agrisystem aspects.

Table 5 shows that vegetables notably had a far greater impact than soil on the combined soil + vegetable risk assessment for lettuce and carrot, however vegetable assessment alone did not always account for risks calculated for the combined agrisystem. As for soil Pb risk indices, there is little comparable literature describing biochar effects on combined soil and vegetable ingestion risks.

### Uncertainty and sensitivity analyses

Supplementary Material; Fig. 1 shows the frequency-probability distributions of soil THQ Monte Carlo Simulations for (A) High Pb conditions, and (B) Low Pb conditions. All THQ values were below the < 1 threshold for significant risk. High Pb conditions reported a greater variability than low Pb conditions. In High Pb conditions, lettuce agrisystem soils reported the greatest variability, and no-crop agrisystems reported the lowest variability. This was also true for Low Pb conditions, with the exception of carrot/control agrisystem soils, which had a notably broad distribution and thus higher uncertainty. This has implications for application and representativeness of risk assessment indices in this agrisystem. In comparing biochar and control treatments, variability was marginally greater for biochar-treated vegetable agrisystem soil THQ in high Pb conditions, though the opposite was true for no-crop agrisystems. These trends are reversed for low Pb conditions.

Supplementary Material; Fig. 2 shows the frequency and probability distributions of the vegetable THQ Monte Carlo Simulations. As reported in Table 4, THQ values differed significantly between vegetable crops and were thus depicted in independent graphs. Vegetable THQ distributions were similar for control and biochar in High Pb conditions. For Low Pb conditions, biochar treatments had a broader distribution, and thus uncertainty, for carrot and garlic. There was a relatively high probability that lettuce THQ may exceed the THQ threshold for significant risk (< 1) in all lettuce agrisystems.

Sensitivity analysis describes the most influential variables in calculating soil and vegetable THQ for each treatment. For soil THQs, ingestion rate had the highest impact on THQ for all treatments, often followed by body weight, BAF, total concentration, and exposure frequency (Supplementary Material; Fig. 3). This pattern was observed for each agrisystem soil, and there were no influencing variable trends observed across high/low Pb soils or between biochar/control applications.

Similar trends were observed for vegetables, however total Pb concentration often had a greater impact on vegetable THQ, compared to soil. Total concentration had a higher influence on lettuce THQ grown in high Pb soils and higher influence of total concentration on carrot THQ grown in biochar treatments (Supplementary Material; Fig. 4).

## Implications for risk assessment

Ultimately, it can be determined that biochar has differential effects on urban grower (soil exposure), vegetable consumer, and combined growers and consumers ingestion risk remediation in soils where properties are minimally altered with biochar application. Using risk indices, biochar has greater Pb remediation potential for soil ingestion, and no remediation potential for those who only consume vegetables. As urban growers are likely both growers and consumers, biochar has some limited remediation potential in low Pb lettuce agrisystems only. Cost–benefit analysis would then be required to determine the usefulness of biochar as a remediation tool in high SOM and alkaline pH urban agrisystems.

This study raises discussion of the following:

- The universal application of biochar as Pb remediation across any contaminated soil, as it may not be effective (or even detrimental) in soils that already possess biochar-similar properties such as high alkalinity and organic matter. These context-specific interactions highlight that biochar should not be treated as a one-size-fits-all amendment; instead, there should be careful alignment between biochar properties and existing soil chemistry to avoid unintended negative outcomes.
- As this study is based on a controlled pot experiment, further investigations into other contributing variables such as crop-soil-biochar interactions in long-term field studies are needed. Alternate exposure risks, such as inhalation, should also be considered in the holistic risk assessment. In addition, logistical differences in agrisystem interactions should be considered, for example, lettuce growers may have more regular contact with soils due to high crop turnover, compared to crops with longer growth periods such as garlic. Further risk assessments should incorporate a holistic view of urban agricultural systems to better quantify grower risks.
- The most accurate measure of site-specific risk assessment between bioaccessibility and risk indices, when evaluating minimally effective remediation measures with confounding variables, such as crop cover. Currently, bioaccessibility is increasingly used in both research and industry soil ingestion risk assessment, however extensive investigations may be needed to determine the appropriateness of each use, particularly where the quantified risk is deemed borderline for action.

## Conclusions

This research investigated biochar potential to reduce human Pb ingestion risks from soil exposure, vegetable consumption, and combined soil exposure and vegetable consumption in four mono-crop agrisystem soils with high organic matter and pH. As anticipated, biochar produced limited and inconsistent changes in soil bioaccessible Pb, vegetable Pb concentrations, and the associated human health risk indices. Biochar generally reduced soil ingestion risks in high Pb no-crop, high Pb and low Pb carrot, and low Pb garlic agrisystems. However biochar had no effect on consumer vegetable ingestion risk indices in any agrisystem, likely due to the confounding influence of plant-root defence mechanisms and biochar nutrient effects on plant health. For the more representative combined exposure scenario, as relevant to urban growers, significant risk reduction from biochar occurred only in low-Pb lettuce agrisystems. Ultimately, these findings indicate that while biochar can contribute to lowering Pb ingestion risks in certain urban agriculture contexts, its effectiveness is highly dependent on crop-soil-biochar interactions, the degree of soil modification achieved, and the choice of risk assessment metrics.

## Supplementary Information

Below is the link to the electronic supplementary material.

Supplementary file1 (DOCX 671 KB)

## Data Availability

The datasets used during the current study are available from the corresponding author upon reasonable request.

## References

[CR1] AFBI. (2025). Agri-Food and Biosciences Institute. https://www.afbini.gov.uk/

[CR2] Albert, H. A., Li, X., Jeyakumar, P., Wei, L., Huang, L., Huang, Q., Kamran, M., Shaheen, S. M., Hou, D., Rinklebe, J., Liu, Z., & Wang, H. (2021). Influence of biochar and soil properties on soil and plant tissue concentrations of Cd and Pb: A meta-analysis. *Science of the Total Environment*. 10.1016/j.scitotenv.2020.142582 10.1016/j.scitotenv.2020.142582 33065502

[CR3] Alloway, B. J. (2013). *Heavy metals in soils: trace metals and metalloids in soils and their bioavailability* (3rd ed.). Springer.

[CR4] Baghaie, A. H., & Fereydoni, M. (2019). The potential risk of heavy metals on human health due to the daily consumption of vegetables. *Environmental Health Engineering and Management,**6*(1), 11–16.

[CR5] Biasioli, M., Grčman, H., Kralj, T., Madrid, F., Díaz-Barrientos, E., & Ajmone-Marsan, F. (2007). Potentially toxic elements contamination in urban soils. *Journal of Environmental Quality,**36*, 70–79. 10.2134/jeq2006.0254 17215214 10.2134/jeq2006.0254

[CR6] Billmann, M., Hulot, C., Pauget, B., Badreddine, R., Papin, A., & Pelfrêne, A. (2023). Oral bioaccessibility of PTEs in soils: A review of data, influencing factors and application in human health risk assessment. *Science of the Total Environment,**896*, Article 165263. 10.1016/J.SCITOTENV.2023.165263 37400023 10.1016/j.scitotenv.2023.165263

[CR7] British Standards Institution. (2006). ISO 11464:2006: Soil quality - Pretreatment of samples for physico-chemical analysis. In BSI. https://www.iso.org/standard/37718.html

[CR8] Caporale, A. G., Porfido, C., Roggero, P. P., Di Palma, A., Adamo, P., Pinna, M. V., Garau, G., Spagnuolo, M., & Castaldi, P. (2023). Long-term effect of municipal solid waste compost on the recovery of a potentially toxic element (PTE)-contaminated soil: PTE mobility, distribution and bioaccessibility. *Environmental Science and Pollution Research,**30*(58), 122858–122874. 10.1007/s11356-023-30831-y 37979102 10.1007/s11356-023-30831-y PMC10724333

[CR9] Cheng, C., Luo, W., Wang, Q., He, L., & Sheng, X. (2020). Combined biochar and metal-immobilizing bacteria reduces edible tissue metal uptake in vegetables by increasing amorphous Fe oxides and abundance of Fe- and Mn-oxidising Leptothrix species. *Ecotoxicology and Environmental Safety,**206*, Article 111189. 10.1016/J.ECOENV.2020.111189 32858328 10.1016/j.ecoenv.2020.111189

[CR10] Cocerva, T., Robb, M., Wong, A., Doherty, R., Newell, J., Ofterdinger, U., Carey, M., Cave, M., & Cox, S. F. (2024). Using oral bioaccessibility measurements to refine risk assessment of potentially toxic elements in topsoils across an urban area. *Ecotoxicology and Environmental Safety,**276*, Article 116293. 10.1016/j.ecoenv.2024.116293 38599155 10.1016/j.ecoenv.2024.116293

[CR82] Du, H., Yin, N., Cai, X., Wang, P., Li, Y., Fu, Y., Sultana, M. S., Sun, G., & Cui, Y. (2020). Lead bioaccessibility in farming and mining soils: The influence of soil properties, types and human gut microbiota. The Science of the Total Environment, 708. 10.1016/J.SCITOTENV.2019.135227 10.1016/j.scitotenv.2019.135227 31812419

[CR11] Ene, A., Sion, A., Stihi, C., Gheboianu, A. I., Basliu, V., Ceoromila, A. M. C., & Gosav, S. (2024). Metal Contamination and Human Health Risk Assessment of Soils from Parks and Playgrounds of an Industrialized Town (Galati, Romania) [pre-print]. 10.20944/PREPRINTS202410.0732.V1

[CR13] Environment Agency. (2009). CLEA Software (Version 1.05) Handbook Better Regulation Science Programme Science report: SC050021/SR4. https://assets.publishing.service.gov.uk/media/5a816e63e5274a2e87dbd977/LIT_10167.pdf

[CR14] Fahr, M., Laplaze, L., Bendaou, N., Hocher, V., El Mzibri, M., Bogusz, D., & Smouni, A. (2013). Effect of lead on root growth. *Frontiers in Plant Science*. 10.3389/fpls.2013.00175 10.3389/fpls.2013.00175 PMC3674728 23750165

[CR15] Fallahizadeh, S., Hosseini gousheh, S. N., Hossaini motlagh, A., Zarei, M., Rahimi, N., & Sadat, S. A. (2025). Health risk assessment of heavy metals in drinking water reservoirs of Yasuj Iran using Monte Carlo simulation and sensitivity analysis. *Journal of Food Composition and Analysis,**148*, Article 108398. 10.1016/j.jfca.2025.108398

[CR16] FAO. (2023). General Standard for Contaminants and Toxins in Food and Feed. International Food Standards, CXS 193-1995. Food and Agriculture Organisation of the United Nations, World Health Organisation. https://www.fao.org/fao-who-codexalimentarius/sh-proxy/en/?lnk=1&url=https%253A%252F%252Fworkspace.fao.org%252Fsites%252Fcodex%252FStandards%252FCXS%2B193-1995%252FCXS_193e.pdf

[CR17] Gomes, F. P., Barreto, M. S. C., Amoozegar, A., & Alleoni, L. R. F. (2022). Immobilization of lead by amendments in a mine-waste impacted soil: Assessing Pb retention with desorption kinetic, sequential extraction and XANES spectroscopy. *Science of the Total Environment,**807*, Article 150711. 10.1016/J.SCITOTENV.2021.150711 34626622 10.1016/j.scitotenv.2021.150711

[CR18] Guerrieri, N., Mazzini, S., & Borgonovo, G. (2024). Food plants and environmental contamination: An update. *Toxics,**12*(5), 365. 10.3390/toxics12050365 38787144 10.3390/toxics12050365 PMC11125986

[CR19] Guo, M., Song, W., & Tian, J. (2020). Biochar-facilitated soil remediation: mechanisms and efficacy variations. *Frontiers in Environmental Science,**8*, Article 521512. 10.3389/FENVS.2020.521512/BIBTEX

[CR20] Gupta, N., Yadav, K. K., Kumar, V., Prasad, S., Cabral-Pinto, M. M. S., Jeon, B. H., Kumar, S., Abdellattif, M. H., & Alsukaibia, A. K. D. (2022). Investigation of heavy metal accumulation in vegetables and health risk to humans from their consumption. *Frontiers in Environmental Science,**10*, Article 791052. 10.3389/FENVS.2022.791052/BIBTEX

[CR21] Habte, G., Mekonen, N., Desse, G., & Kassa, G. (2023). Heavy metal contamination and health risk assessment of horticultural crops in two sub-cities of Addis Ababa. *Ethiopia. Toxicology Reports,**11*, 420. 10.1016/J.TOXREP.2023.09.002 38021469 10.1016/j.toxrep.2023.09.002 PMC10630556

[CR22] Harries, N., Quint, M., Firth, S., Stutt, E., Bull, S., Pease, C., Hart, A., Macarthur, R., Kennedy, M., & Moreby, S. (2014). SP1010 – Development of Category 4 Screening Levels for Assessment of Land Affected by Contamination. https://www.claire.co.uk/projects-and-initiatives/category-4-screening-levels

[CR23] Hegab, R. H., Eissa, D., Abou-Shady, A., & Abdelmottaleb, O. (2016). Effect of biochar addition on soil properties and carrot productivity grown in polluted soils. *Egyptian Journal of Desert Research,**66*(2), 327–350.

[CR24] Hinds, J. (2023). Soil Remediation, Soil Health and Nutrient Management . https://www.sare.org/publications/best-practices-for-the-sustainable-urban-farm/soil-remediation-soil-health-and-nutrient-management/

[CR25] Hinks, R., Nicolini, G., Ritchie, P., Sutherland, J., Taylor, A., Ward, A., Wheeler, A., & Williamson, D. (2016). Veg Facts: A briefing by the Food Foundation. www.foodfoundation.org.uk/peasplease

[CR27] Janus, A., Waterlot, C., Heymans, S., Deboffe, C., Douay, F., & Pelfrêne, A. (2018). Do biochars influence the availability and human oral bioaccessibility of Cd, Pb, and Zn in a contaminated slightly alkaline soil? *Environmental Monitoring and Assessment*. 10.1007/s10661-018-6592-8 10.1007/s10661-018-6592-8 29541923

[CR28] Jeffries, J., & Martin, I. (2009). SC050021/SR3: Updated technical background to the CLEA model . www.environment-agency.gov.uk

[CR29] Khan, A. Z., Ding, X., Khan, S., Ayaz, T., Fidel, R., & Khan, M. A. (2020b). Biochar efficacy for reducing heavy metals uptake by cilantro (Coriandrum sativum) and spinach (Spinaccia oleracea) to minimize human health risk. *Chemosphere*. 10.1016/j.chemosphere.2019.125543 10.1016/j.chemosphere.2019.125543 32050340

[CR30] Khan, A. Z., Khan, S., Ayaz, T., Brusseau, M. L., Khan, M. A., Nawab, J., & Muhammad, S. (2020c). Popular wood and sugarcane bagasse biochars reduced uptake of chromium and lead by lettuce from mine-contaminated soil. *Environmental Pollution,**263*, Article 114446. 10.1016/J.ENVPOL.2020.114446 32283452 10.1016/j.envpol.2020.114446 PMC7654435

[CR31] Khan, A., Khan, S., Lei, M., Alam, M., Khan, M. A., & Khan, A. (2020a). Biochar characteristics, applications and importance in health risk reduction through metal immobilization. *Environmental Technology & Innovation,**20*, Article 101121. 10.1016/J.ETI.2020.101121

[CR32] Kicińska, A., & Wikar, J. (2024). Health risk associated with soil and plant contamination in industrial areas. *Plant and Soil,**498*, 295–323. 10.1007/s11104-023-06436-2

[CR33] Kim, I., & Kim, K. (2019). Estimation of water footprint for major agricultural and livestock products in Korea. *Sustainability,**11*(10), 2980. 10.3390/su11102980

[CR34] Knoblauch, C., Priyadarshani, R., H, S., Haefele, S. M., Schröder, N., & Pfeiffer, E.-M. (2021). Impact of biochar on nutrient supply, crop yield and microbial respiration on sandy soils of northern Germany. *European Journal of Soil Science*. 10.1111/ejss.13088

[CR35] Kumar, A., Kumar, A., Cabral-Pinto, M., Chaturvedi, A. K., Shabnam, A. A., Subrahmanyam, G., Mondal, R., Gupta, D. K., Malyan, S. K., Kumar, S. S., Khan, S. A., & Yadav, K. K. (2020). Lead toxicity: Health hazards, influence on food chain, and sustainable remediation approaches. *International Journal of Environmental Research and Public Health*. 10.3390/ijerph17072179. MDPI AG. 10.3390/ijerph17072179 PMC7177270 32218253

[CR36] Lehmann, J., & Joseph, S. (2015). *Biochar for environmental management: Science, technology and implementation* (2nd ed.). Routledge.

[CR37] Li, G., Chi, H., Hou, Y., Williams, P. N., Liu, Z., & Cai, C. (2024). Accurate bioaccessibility assessment of soil heavy metals by combining their speciation and in vitro model with human gut microbiota. *Environmental Sciences Europe*. 10.1186/s12302-024-01038-w

[CR38] Lian, W., Shi, W., Tian, S., Gong, X., Yu, Q., Lu, H., Liu, Z., Zheng, J., Wang, Y., Bian, R., Li, L., & Pan, G. (2023). Preparation and application of biochar from co-pyrolysis of different feedstocks for immobilization of heavy metals in contaminated soil. *Waste Management,**163*, 12–21. 10.1016/J.WASMAN.2023.03.022 36989826 10.1016/j.wasman.2023.03.022

[CR39] Liu, S., Cen, B., Yu, Z., Qiu, R., Gao, T., & Long, X. (2025). The key role of biochar in amending acidic soil: Reducing soil acidity and improving soil acid buffering capacity. *Biochar*. 10.1007/s42773-025-00432-8

[CR40] Lu, J., Ma, J., Wang, B., Ogino, K., Si, H., & Li, Y. (2025). Study on mechanism of biochar improving acid soil: Multi-scale experiment and numerical simulation. *Journal of Environmental Management,**389*, Article 126083. 10.1016/j.jenvman.2025.126083 40480113 10.1016/j.jenvman.2025.126083

[CR41] McBride, M. B., Shayler, H. A., Spliethoff, H. M., Mitchell, R. G., Marquez-Bravo, L. G., Ferenz, G. S., Russell-Anelli, J. M., Casey, L., & Bachman, S. (2014). Concentrations of lead, cadmium and barium in urban garden-grown vegetables: The impact of soil variables. *Environmental Pollution,**194*, 254–261. 10.1016/j.envpol.2014.07.036 25163429 10.1016/j.envpol.2014.07.036 PMC4175907

[CR42] Medyńska-Juraszek, A., Ćwieląg-Piasecka, I., Weber, J., & Bekier, J. (2013). Efficiency of biochar soil amendments for reducing copper and zinc mobility and uptake by Red Clover (Trifolium pretense L.). BCD 2013 “Biochars, Composts, and Digestates” Production, Characterization, Regulation, Marketing, Uses and Environmental Impact. https://www.researchgate.net/publication/290430973_Efficiency_of_biochar_soil_amendments_for_reducing_copper_and_zinc_mobility_and_uptake_by_Red_Clover_Trifolium_pretense_L

[CR43] Medyńska-Juraszek, A., Bednik, M., & Chohura, P. (2020). Assessing the influence of compost and biochar amendments on the mobility and uptake of heavy metals by green leafy vegetables. *International Journal of Environmental Research and Public Health*. 10.3390/ijerph17217861 10.3390/ijerph17217861 PMC7662399 33121066

[CR44] Mehta, N., Cipullo, S., Cocerva, T., Coulon, F., Dino, G. A., Ajmone-Marsan, F., Padoan, E., Cox, S. F., Cave, M. R., & De Luca, D. A. (2020). Incorporating oral bioaccessibility into human health risk assessment due to potentially toxic elements in extractive waste and contaminated soils from an abandoned mine site. *Chemosphere*. 10.1016/j.chemosphere.2020.126927 10.1016/j.chemosphere.2020.126927 32417510

[CR45] Mianeh, H. Y., Amiri, L., Jafari, A., & Nourozi, N. (2025). Health risk assessment via Monte Carlo simulation and sensitivity analysis for fluoride and nitrate content in bottled waters consumed in Kermanshah city, Iran. *Scientific Reports*. 10.1038/s41598-025-89439-6 10.1038/s41598-025-89439-6 PMC11811224 39929929

[CR46] Mielke, H. W., Gonzales, C. R., Smith, M. K., & Mielke, P. W. (2000). Quantities and associations of lead, zinc, cadmium, manganese, chromium, nickel, vanadium, and copper in fresh Mississippi Delta alluvium and New Orleans alluvial soils. *Science of the Total Environment,**246*(2–3), 249–259. 10.1016/s0048-9697(99)00462-3 10696726 10.1016/s0048-9697(99)00462-3

[CR47] Moazamnia, M., Sadeghfam, S., Jabraili-Andariyan, N., Nadiri, A. A., Mirabbasi, R., & Noori, R. (2024). Probabilistic human health risk assessment for arsenic, nickel and lead exposures based on two-dimensional Monte Carlo simulation. *Groundwater for Sustainable Development*. 10.1016/j.gsd.2024.101312

[CR49] Mousavi, S. M., Brodie, G., Payghamzadeh, K., Raiesi, T., & Strivastava, A. K. (2022). Lead bioavailability in the environment: Its exposure and and effects. *Journal of Advances in Environmental Health Research,**10*(1), 1–14. 10.32598/JAEHR.10.1.1256

[CR50] Müller, A., Österlund, H., Marsalek, J., & Viklander, M. (2020). The pollution conveyed by urban runoff: A review of sources. *Science of the Total Environment,**709*, Article 136125. 10.1016/J.SCITOTENV.2019.136125 31905584 10.1016/j.scitotenv.2019.136125

[CR51] Nag, R., & Cummins, E. (2022). Human health risk assessment of lead (Pb) through the environmental-food pathway. *Science of the Total Environment,**810*, Article 151168. 10.1016/J.SCITOTENV.2021.151168 34710405 10.1016/j.scitotenv.2021.151168

[CR52] Nawab, J., Ghani, J., Khan, S., & Xiaoping, W. (2018). Minimizing the risk to human health due to the ingestion of arsenic and toxic metals in vegetables by the application of biochar, farmyard manure and peat moss. *Journal of Environmental Management,**214*, 172–183. 10.1016/J.JENVMAN.2018.02.093 29525749 10.1016/j.jenvman.2018.02.093

[CR53] Netherway, P., Reichman, S. M., Laidlaw, M., Scheckel, K., Pingitore, N., Gascó, G., Méndez, A., Surapaneni, A., & Paz-Ferreiro, J. (2019). Phosphorus-rich biochars can transform lead in an urban contaminated soil. *Journal of Environmental Quality,**48*, 1091–1099. 10.2134/jeq2018.09.0324 31589692 10.2134/jeq2018.09.0324

[CR54] Newell, J., Cox, S. F., & Doherty, R. (2025). Oral bioaccessibility trends for As, Cd, Cr, Ni, and Pb in vegetables grown in contaminated soils: A systematic review. *Urban Agriculture and Regional Food Systems*. 10.1002/UAR2.70009

[CR55] Nowar, A., Islam, M. H., Islam, S., Jubayer, A., & Nayan, M. M. (2024). A systematic review on heavy metals contamination in Bangladeshi vegetables and their associated health risks. *Frontiers in Environmental Science,**12*, Article 1425286. 10.3389/FENVS.2024.1425286 10.1177/11786302241309280 PMC11672373 39735426

[CR56] Pandey, A. K., Shrivastava, A., Vashishth, R., & Chauhan, O. (2025). Structure and composition of fruits and vegetables. *Fruits and Vegetables Technologies*. 10.1007/978-981-96-8433-5_1

[CR57] Park, J. H., Choppala, G. K., Bolan, N. S., Chung, J. W., & Chuasavathi, T. (2011). Biochar reduces the bioavailability and phytotoxicity of heavy metals. *Plant and Soil,**348*(1–2), 439–451. 10.1007/S11104-011-0948-Y

[CR58] Paustenbach, D. J. (2000). The practice of exposure assessment: A state-of-the-art review. *Journal of Toxicology and Environmental Health - Part B: Critical Reviews,**3*(3), 179–291. 10.1080/10937400050045264 10911984 10.1080/10937400050045264

[CR59] Pavlíková, D., Zemanová, V., & Pavlík, M. (2023). Health risk and quality assessment of vegetables cultivated on soils from a heavily polluted old mining area. *Toxics,**11*(7), 583. 10.3390/toxics11070583 37505549 10.3390/toxics11070583 PMC10384379

[CR60] Payen, F. T., Evans, D. L., Falagán, N., Hardman, C. A., Kourmpetli, S., Liu, L., Marshall, R., Mead, B. R., & Davies, J. A. C. (2022). How much food can we grow in urban areas? Food production and crop yields of urban agriculture: A meta-analysis. *Earth’s Future,**10*(8), Article e2022EF002748. 10.1029/2022EF002748 10.1029/2022EF002748 PMC9540868 36246543

[CR61] Poggio, L., Vrščaj, B., Schulin, R., Hepperle, E., & Ajmone Marsan, F. (2009). Metals pollution and human bioaccessibility of topsoils in Grugliasco (Italy). *Environmental Pollution,**157*, 680–689. 10.1016/j.envpol.2008.08.009 18835073 10.1016/j.envpol.2008.08.009

[CR62] Pourrut, B., Shahid, M., Dumat, C., Winterton, P., & Pinelli, E. (2011). Lead uptake, toxicity, and detoxification in plants. *Reviews of Environmental Contamination and Toxicology,**213*, 113–136. 10.1007/978-1-4419-9860-6_4 21541849 10.1007/978-1-4419-9860-6_4

[CR63] Qasim, B., Razzak, A. A., & Rasheed, R. T. (2021). Effect of biochar amendment on mobility and plant uptake of Zn, Pb and Cd in contaminated soil. *IOP Conference Series: Earth and Environmental Science*. 10.1088/1755-1315/779/1/012082

[CR64] Ritchie, H., & Roser, M. (2022). Lead Pollution. *Our World in Data*. https://ourworldindata.org/lead-pollution

[CR65] Salam, A., Bashir, S., Khan, I., Hussain, Q., Gao, R., & Hu, H. (2019). Biochar induced Pb and Cu immobilization, phytoavailability attenuation in Chinese cabbage, and improved biochemical properties in naturally co-contaminated soil. *Journal of Soils and Sediments,**19*(5), 2381–2392. 10.1007/s11368-019-02250-5

[CR66] Shaheen, N., Irfan, N. M., Khan, I. N., Islam, S., Islam, M. S., & Ahmed, M. K. (2016). Presence of heavy metals in fruits and vegetables: Health risk implications in Bangladesh. *Chemosphere,**152*, 431–438. 10.1016/J.CHEMOSPHERE.2016.02.060 27003365 10.1016/j.chemosphere.2016.02.060

[CR67] Tan, G., & Yu, H. Q. (2023). Rethinking biochar: Black gold or not? *Nature Reviews Materials,**9*(1), 4–5. 10.1038/s41578-023-00634-1

[CR68] United Nations. (2019). World Urbanization Prospects: The 2018 Revision (ST/ESA/SER.A/420). https://population.un.org/wup/Publications/Files/WUP2018-Report.pdf

[CR83] Uzu, G., Sobanska, S., Sarret, G., Muñoz, M., & Dumat, C. (2010). Foliar lead uptake by lettuce exposed to atmospheric fallouts. Environmental Science & Technology, 44(3), 1036–1042. 10.1021/ES902190U 10.1021/es902190u 20063891

[CR69] Vannini, A., Bianchi, E., Avi, D., Damaggio, N., Di Lella, L. A., Nannoni, F., Protano, G., & Loppi, S. (2021). Biochar amendment reduces the availability of Pb in the soil and its uptake in lettuce. *Toxics*. 10.3390/toxics9100268 10.3390/toxics9100268 PMC8539867 34678964

[CR70] Vienne, A., Newell, J., Roussard, J., Doherty, R., Cox, S. F., Lyons, G., & Vicca, S. (2026). Effects of basalt and biochar addition on base cations and trace metals in plants and soil in an urban field trial. *Biogeosciences,**23*(4), 1681–1695. 10.5194/bg-23-1681-2026

[CR71] Wang, G., Tariq, M., Liang, W., Wan, J., Peng, C., Zhang, W., Cao, X., & Lou, Z. (2022). A comparative and modeled approach for three biochar materials in simultaneously preventing the migration and reducing the bioaccessibility of heavy metals in soil: Revealing immobilization mechanisms. *Environmental Pollution,**309*, Article 119792. 10.1016/J.ENVPOL.2022.119792 35863701 10.1016/j.envpol.2022.119792

[CR72] Wani, A. L., Ara, A., & Usmani, J. A. (2015). Lead toxicity: A review. *Interdisciplinary Toxicology,**8*(2), 55. 10.1515/INTOX-2015-0009 27486361 10.1515/intox-2015-0009 PMC4961898

[CR73] Weaver, J. E., & Bruner, W. E. (1927). *Root development of vegetable crops* (1st ed.). McGraw-Hill Book Company, Inc.

[CR81] Wragg, J., & Cave, M. R. (2003). In-vitro methods for the measurement of the oral bioaccessibility of selected metals and metalloids in soils : a review of guidance and selected industry practices. Environment Agency

[CR74] Wragg, J., Cave, M., Taylor, H., Basta, N., Brandon, E., Casteel, S., Gron, C., Oomen, A., & Van De Wiele, T. (2009). Inter-laboratory trial of a Unified Bioaccessibility Procedure. www.bgs.ac.uk/gsni/10.1016/j.scitotenv.2011.05.019 21703664

[CR75] Yang, Z., Fang, Z., Zheng, L., Cheng, W., Tsang, P. E., Fang, J., & Zhao, D. (2016). Remediation of lead contaminated soil by biochar-supported nano-hydroxyapatite. *Ecotoxicology and Environmental Safety,**132*, 224–230. 10.1016/J.ECOENV.2016.06.008 27337496 10.1016/j.ecoenv.2016.06.008

[CR76] Zhang, D., Li, T., Wu, X., & Wang, Y. (2021). Effect of amendments (lime–zeolite–biochar) on the immobilization of Cd and Pb in a contaminated acidic soil. *IOP Conference Series: Earth and Environmental Science,**742*(1), Article 012016. 10.1088/1755-1315/742/1/012016

[CR77] Zhang, S., Gu, W., Bai, J., Dong, B., Zhao, J., & Zhuang, X. (2023). Fate and health risk assessment of heavy metals in *Brassica chinensis L.* (pak-choi) and soil amended by sludge-based biochar. *Environmental Science and Pollution Research International,**30*(3), 5621–5633. 10.1007/S11356-022-22358-5 35980524 10.1007/s11356-022-22358-5

[CR78] Zhao, W., Liao, Y., Zhou, S., & Zhou, B. (2022). Ecological remediation strategy for urban brownfield renewal in Sichuan Province, China: A health risk evaluation perspective. *Scientific Reports,**12*(1), 1–14. 10.1038/s41598-022-08268-z 35277571 10.1038/s41598-022-08268-z PMC8917217

[CR79] Zheng, X., Zhang, Z., Chen, J., Liang, H., Chen, X., Qin, Y., Shohag, M. J. I., Wei, Y., & Gu, M. (2022). Comparative evaluation of in vivo relative bioavailability and in vitro bioaccessibility of arsenic in leafy vegetables and its implication in human exposure assessment. *Journal of Hazardous Materials*. 10.1016/j.jhazmat.2021.126909 10.1016/j.jhazmat.2021.126909 34454790

[CR80] Zou, Z., Fan, L., Li, X., Dong, C., Zhang, L., Zhang, L., Fu, J., Han, W., & Yan, P. (2021). Response of plant root growth to biochar amendment: A meta-analysis. *Agronomy,**11*(12), Article 2442. 10.3390/AGRONOMY11122442/S1

