# Supplementary material for: Digestate biochar effects on soil Pb bioaccessibility, crop Pb concentrations, and human health risk in urban vegetable agroecosystems
Source: Environ Geochem Health. 2026 May 28;48(8):376. doi: 10.1007/s10653-026-03269-7 (PMC13219143; doi:10.1007/s10653-026-03269-7)
Supplement: Supplementary file 1 — Supplementary file1 (DOCX 671 KB) [file 10653_2026_3269_MOESM1_ESM.docx]

Title: Digestate biochar effects on soil Pb bioaccessibility, crop Pb concentrations, and human health risk in urban vegetable agroecosystems

Authors and affiliation: Jennifer Newell ^a*^, Rory Doherty ^a^, Gary Lyons^b^, Siobhan F. Cox^a^

^a^ School of Natural and Built Environment, Queen’s University Belfast, Belfast BT9 5AG, UK

^b^Agri-Environment Branch, Agri-Food and Biosciences Institute, Large Park, Hillsborough, BT26 6DR, UK

* Corresponding author, e-mail: [jnewell07@qub.ac.uk](mailto:jnewell07@qub.ac.uk), +44(0)2890974746

Table 1: Variable parameters used for the soil THQ Monte Carlo Simulations. NC_ = No crop, L_ = Lettuce, C_ = Carrot, G_ = Garlic, _HNB = High Pb/Control, _HB = High Pb/Biochar, _LNB = Low Pb/ Control, _LB = Low Pb/Biochar.

| Soil | Total Pb concentration / mg/kg | Bioaccessible Fraction (BAF) / % | Exposure Frequency (EF) / day | Ingestion Rate (IR) / mg/day | Body Weight (BW) / kg |
| --- | --- | --- | --- | --- | --- |
| NC_HNB | 466.3-552.6 | 42.22-51.88 | 180 - 365 | 50 - 200 | 57.59 - 76.27 |
| NC_HB | 568.1-621.0 | 34.60-38.33 |  |  |  |
| NC_LNB | 184.2-281.3 | 26.03-37.28 |  |  |  |
| NC_LB | 265.8-274.6 | 28.33-31.70 |  |  |  |
| L_HNB | 501.1-648.1 | 47.23-58.87 |  |  |  |
| L_HB | 539.4-591.2 | 47.52-63.16 |  |  |  |
| L_LNB | 247.0-261.6 | 47.75-55.25 |  |  |  |
| L_LB | 212.2-226.3 | 46.39-62.00 |  |  |  |
| C_HNB | 610.2-674.3 | 41.43-46.84 |  |  |  |
| C_HB | 509.1-538.3 | 45.37-51.54 |  |  |  |
| C_LNB | 259.2-489.3 | 39.21-66.36 |  |  |  |
| C_LB | 219.3-301.6 | 31.73-40.36 |  |  |  |
| G_HNB | 464.5-597.6 | 45.89-56.24 |  |  |  |
| G_HB | 446.1-517.2 | 44.65-59.18 |  |  |  |
| G_LNB | 275.8-206.6 | 46.35-61.57 |  |  |  |
| G_LB | 201.6-247.8 | 34.72-45.44 |  |  |  |

Table 2: Variable parameters used for the vegetable THQ Monte Carlo Simulations. L_ = Lettuce, C_ = Carrot, G_ = Garlic, _HNB = High Pb/Control, _HB = High Pb/Biochar, _LNB = Low Pb/ Control, _LB = Low Pb/Biochar.

| Vegetable | Total Pb Concentrations / mg/kg | Exposure Frequency (EF) / day | Ingestion Rate (IR) / mg/day | Body Weight (BW) / kg |
| --- | --- | --- | --- | --- |
| L_HNB | 4.29-10.67 | 180 - 365 | 0 - 400 | 57.59 - 76.27 |
| L_HB | 5.24-12.72 |  |  |  |
| L_LNB | 11.16-16.70 |  |  |  |
| L_LB | 7.72-9.96 |  |  |  |
| C_HNB | 1.71-2.14 |  |  |  |
| C_HB | 0.95-3.07 |  |  |  |
| C_LNB | 0.44-0.46 |  |  |  |
| C_LB | 0.47-1.90 |  |  |  |
| G_HNB | 0.27-0.39 |  |  |  |
| G_HB | 0.20-0.45 |  |  |  |
| G_LNB | 0.05-0.15 |  |  |  |
| G_LB | 0.13-0.23 |  |  |  |

**
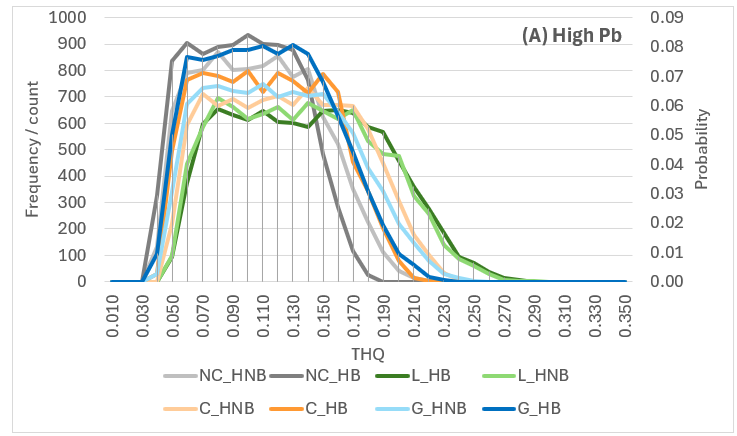
**

**
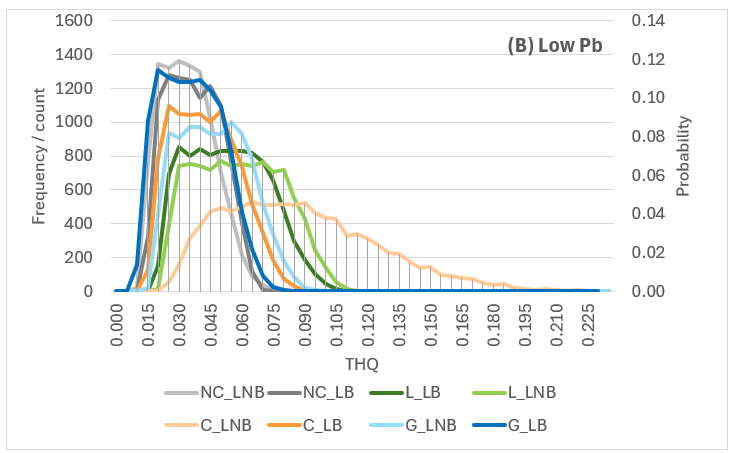
**

**Fig 1** Soil THQ Monte Carlo frequency / probability distributions for (A) High Pb treatments and (B) Low Pb treatments. NC_ = No crop, L_ = Lettuce, C_ = Carrot, G_ = Garlic, _HNB = High Pb/Control, _HB = High Pb/Biochar, _LNB = Low Pb/ Control, _LB = Low Pb/Biochar

|  |  |
| --- | --- |
|  |  |
|  |  |

Fig. 2 Vegetable THQ Monte Carlo frequency / probability distributions. NC_ = No crop, L_ = Lettuce, C_ = Carrot, G_ = Garlic, _HNB = High Pb/Control, _HB = High Pb/Biochar, _LNB = Low Pb/ Control, _LB = Low Pb/Biochar


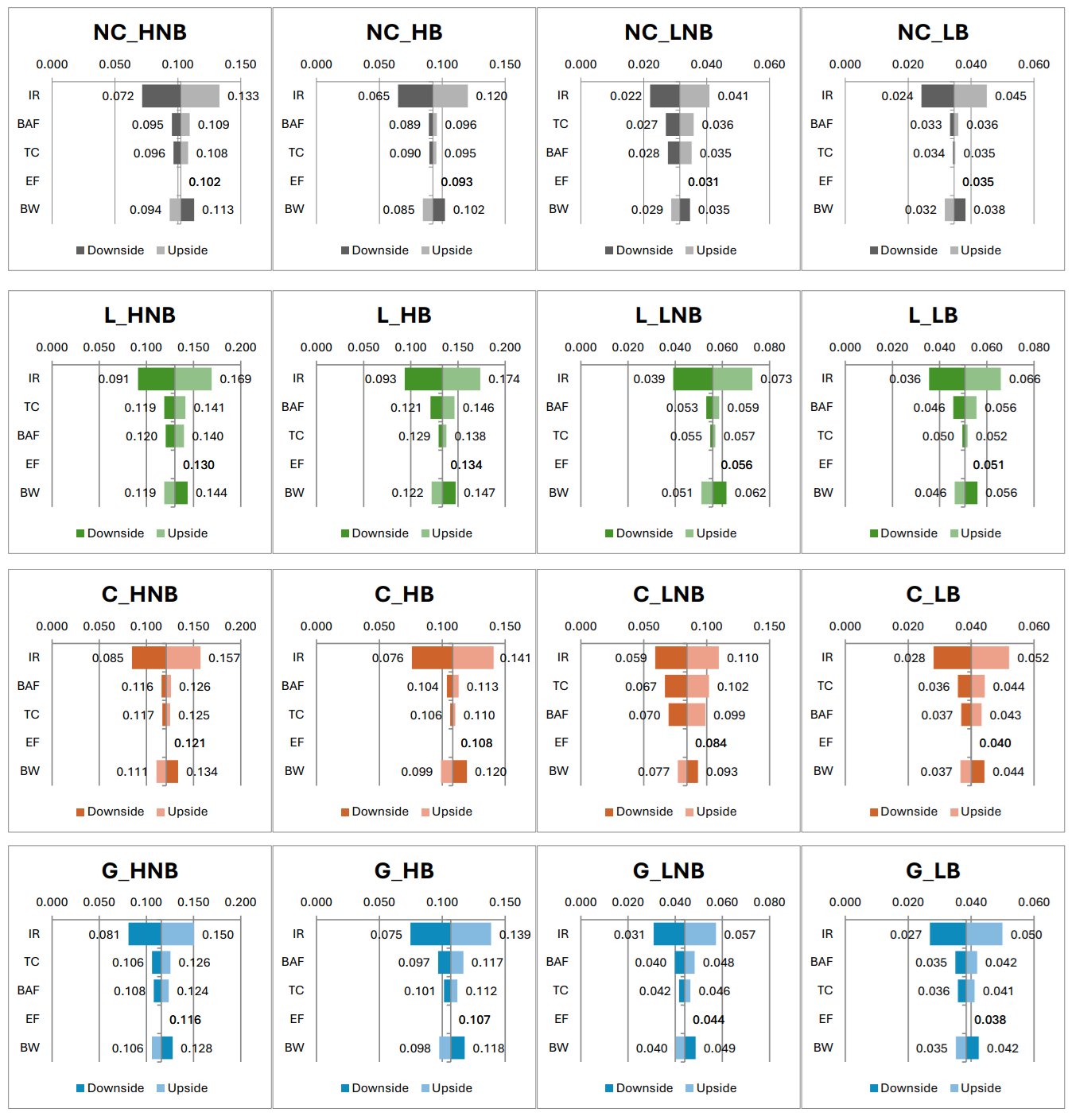


Fig. 3 Sensitivity analysis for soil high/low Pb, biochar/control, crop agrisystem treatments. NC_ = No crop, L_ = Lettuce, C_ = Carrot, G_ = Garlic, _HNB = High Pb/Control, _HB = High Pb/Biochar, _LNB = Low Pb/ Control, _LB = Low Pb/Biochar. IR = Ingestion Rate, BAF = Bioaccessible Fraction; TC = Total Concentration; EF = Exposure Frequency; BW = Body Weight


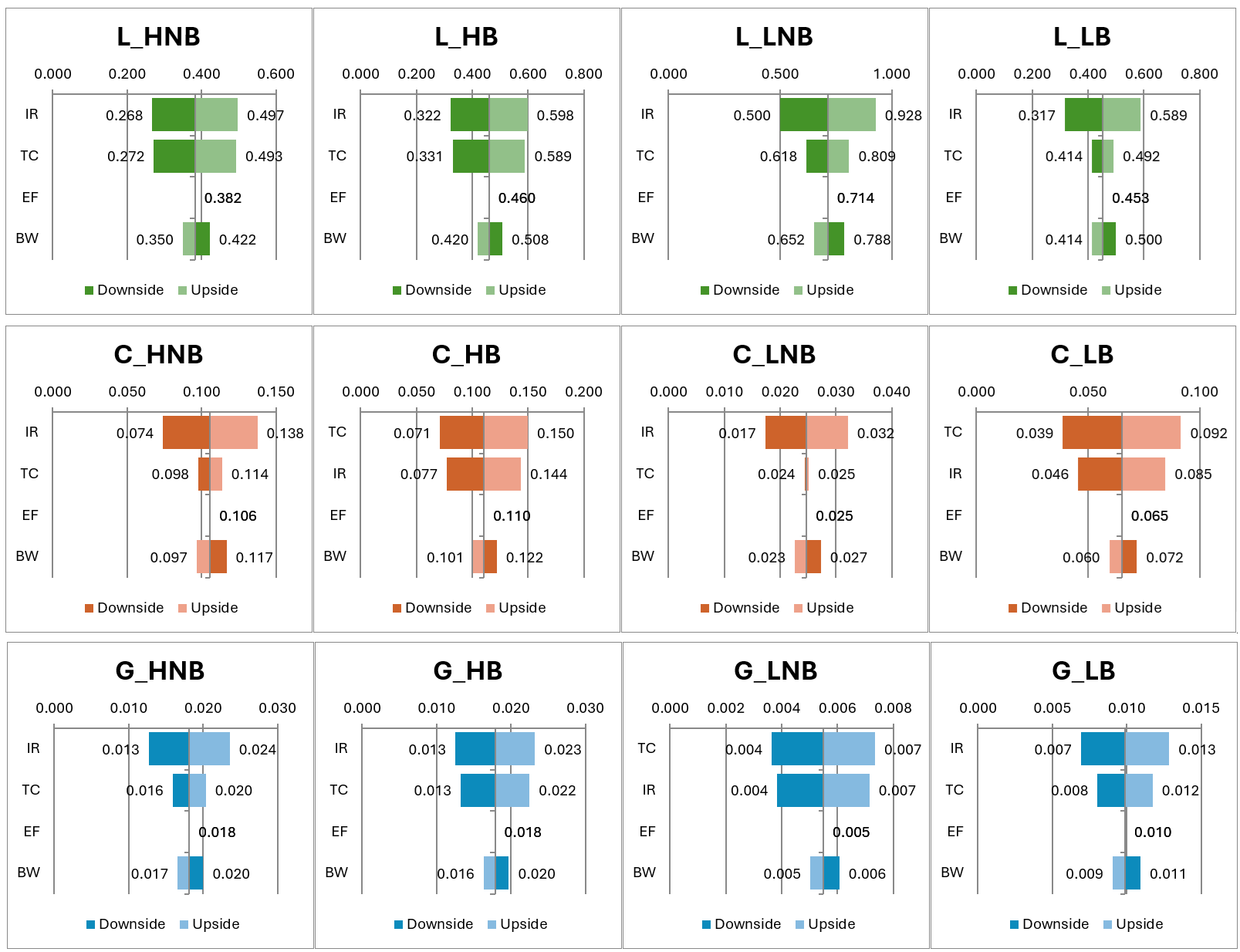


Fig. 4 Sensitivity analysis for soil high/low Pb, biochar/control, lettuce carrot and garlic crops. L_ = Lettuce, C_ = Carrot, G_ = Garlic, _HNB = High Pb/Control, _HB = High Pb/Biochar, _LNB = Low Pb/ Control, _LB = Low Pb/Biochar. IR = Ingestion Rate, BAF = Bioaccessible Fraction; TC = Total Concentration; EF = Exposure Frequency; BW = Body Weight
